# Chiral nanoprobes for targeting and long-term imaging of the Golgi apparatus†
†Electronic supplementary information (ESI) available. See DOI: 10.1039/c7sc01316g



**DOI:** 10.1039/c7sc01316g

**Published:** 2017-07-13

**Authors:** Rong Sheng Li, Peng Fei Gao, Hong Zhi Zhang, Lin Ling Zheng, Chun Mei Li, Jian Wang, Yuan Fang Li, Feng Liu, Na Li, Cheng Zhi Huang

**Affiliations:** a Key Laboratory of Luminescent and Real-Time Analytical Chemistry (Southwest University) , Ministry of Education , College of Pharmaceutical Sciences , Southwest University , Chongqing 400716 , China . Email: chengzhi@swu.edu.cn ; Email: wj123456@swu.edu.cn; b College of Chemistry and Chemical Engineering , Southwest University , Chongqing 400715 , China; c Beijing National Laboratory for Molecular Sciences (BNLMS) , Key Laboratory of Bioorganic Chemistry and Molecular Engineering of Ministry of Education , Institute of Analytical Chemistry , College of Chemistry and Molecular Engineering , Peking University , Beijing , 100871 , China . Email: lina@pku.edu.cn

## Abstract

The Golgi apparatus is an essential subcellular organelle. Targeting and monitoring the Golgi change at the single-cell level over a long time scale are critical but are challenges that have not yet been tackled. Inspired by the precise Golgi positioning ability of galactosyltransferase and protein kinase D, due to their cysteine residues, we developed a method for long-term Golgi imaging. Fluorescent molecules, carbon quantum dots (CQDs) and silica nanoparticles could target the Golgi when they are modified with l-cysteine. l-Cysteine-rich chiral carbon quantum dots (LC-CQDs), which have the benefits of a high Golgi specificity from l-cysteine and excellent photostability and biocompatibility from the CQDs, are proven to be highly suitable for long-term *in situ* imaging of the Golgi. Investigation of the mechanism showed that free thiol groups and the l-type stereo configuration of LC-CQDs are essential for specific targeting of the Golgi. With the aid of the as-prepared LC-CQDs, the dynamic changes of the Golgi in the early stage of viral infection were visualized. The Golgi targeting and imaging strategy used in this work is beneficial for Golgi-targeted drug delivery and early diagnosis and therapy of Golgi diseases.

## Abstract 2

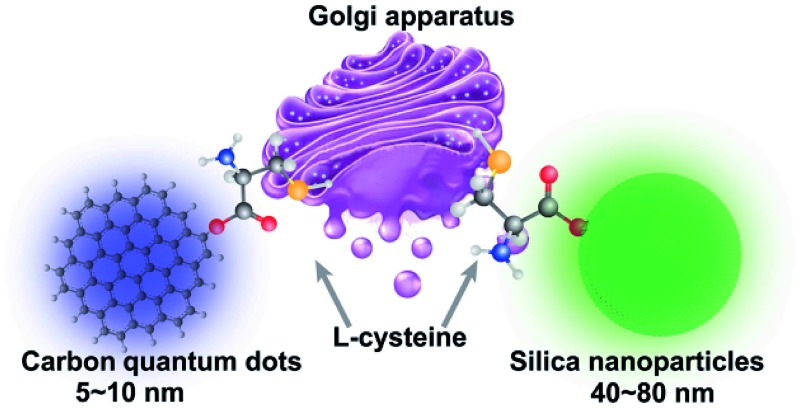
The targeting and long-term imaging of the Golgi apparatus have been realized *via* l-cysteine functionalized nanoprobes.

## Introduction

Subcellular targeting strategies have redefined the frontier of life processes as well as drug design.^1,2^ As a eukaryotic organelle, the Golgi apparatus is essential for biogenesis, secretion, and intracellular distribution of a wide range of macromolecules.^3^ It has been reported that morphological changes of the Golgi are related to external stimuli,^4^ thus can effectively reflect the physiological state of cells. With the development of the membrane fusion method using *N*-[7-(4-nitrobenzo-2-oxa-1,3-diazole)]-6-aminocaproyl sphingosine (NBD C_6_-ceramide) for exploring the sphingolipid transport and metabolism mechanism,^5^ ceramide analogues have become one of the most widely used commercial dyes for labeling the Golgi.^6^ Due to the lack of specificity of ceramide analogues, however, the plasma membranes as well as the mitochondria are also labeled eventually besides the Golgi apparatus. Other Golgi stain approaches,^7,8^ using antibodies, for example, also lack long-term imaging ability, making it difficult to observe real-time changes of the Golgi at the single-cell level under continuous laser excitation. Therefore, it is highly desirable to design and develop new optical probes for the Golgi with a prolonged imaging ability.

Fluorescent carbon quantum dots (CQDs), since their first discovery in 2004,^9^ have been found to be physicochemically and photochemically stable without photobleaching, making them a star family in the biosensing and bioimaging fields.^10–13^ However, bare CQDs are usually weakly fluorescent and it is hard to attach functional groups on the surface.^14,15^ Regardless of continuous efforts to circumvent the problems,^16^ challenges still remain to engineer CQDs with desirable biosensing and labeling properties, such as a high quantum yield and preferable selectivity for the long-term and real-time monitoring of targets of interest.^17^

Herein, we report a novel type of CQD for the long-term *in situ* imaging of the Golgi apparatus. It has been reported that galactosyltransferase and protein kinase D are capable of anchoring in the Golgi apparatus *via* their cysteine residues or cysteine rich domain,^18,19^ which inspires us to combine the principle of the Golgi localization of proteins and carbon nanotechnology to develop an optical probe for Golgi targeting and imaging. We synthesize novel fluorescent CQDs with abundant cysteine residues and an l-type spatial structure using a pyrolysis method with citric acid and l-cysteine as the carbon sources and controlling the pyrolysis temperature. The as-prepared LC-CQDs exhibit excellent long-term Golgi targeting and imaging capabilities that could be attributed to their high quantum yield (68%) and photostability as well as their good biocompatibility. This dependence of the targeting of the Golgi on l-cysteine is further proven by using cysteine modified fluorophores and silica nanoparticles. This study provides an effective method for Golgi targeting and imaging over a long time scale, which may be applied in drug-delivery and therapy, as well as in evaluating disease progression occurring within the Golgi.

## Results and discussion

### Characterization of l-cysteine-rich CQDs (LC-CQDs)

**Fig. 1:**
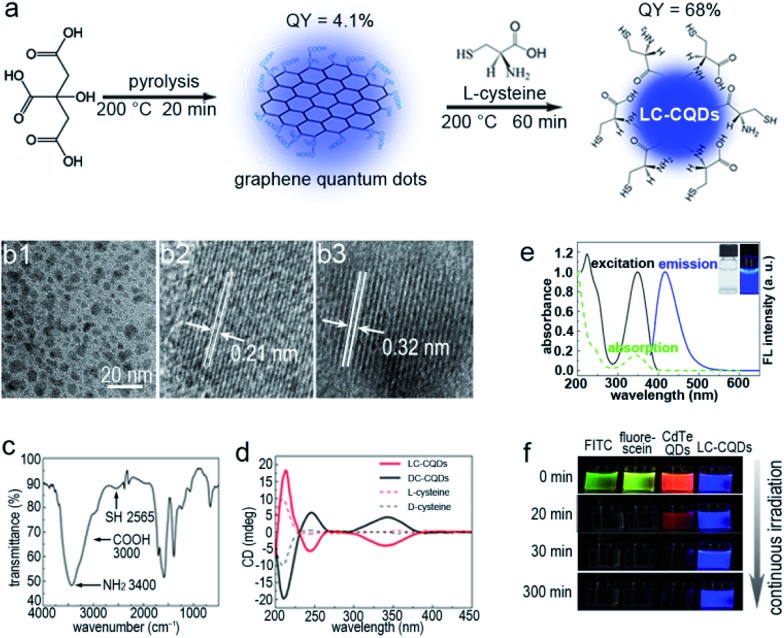
Synthetic route and characterization of the l-cysteine-rich chiral carbon quantum dots (LC-CQDs). (a) Synthetic route of the LC-CQDs by heating citric acid and l-cysteine. The fluorescence quantum yield (QY) of the LC-CQDs is 68%. (b1) HRTEM image of the LC-CQDs. (b2 and b3) Lattice spacing of a typical single LC-CQD. (c) FTIR spectrum. (d) Circular dichroism spectra of both LC-CQDs and DC-CQDs. The DC-CQDs were prepared by heating citric acid and d-cysteine. (e) Fluorescence spectra (solid lines) and UV/Vis absorption spectrum (dotted line) of the LC-CQDs. Inset: Photographs of the LC-CQDs under illumination by white light (left) and UV (365 nm) light (right). (f) Photostability of fluorescein isothiocyanate (FITC), fluorescein, CdTe QDs, and the LC-CQD aqueous solution under continuous irradiation using a 280 W xenon lamp.

The as-prepared LC-CQDs eventually are 8.5 ± 3.5 nm in diameter and are highly fluorescent with an absolute quantum yield of 68% (Fig. 1a and b and S1–S4†). The free thiol group of l-cysteine is well preserved on the surface of the LC-CQDs, as indicated by the vibrational frequency (–SH, 2565 cm^–1^) of the free thiol groups (Fig. 1c and S5–S10).^20^ The average number of l-cysteine residues on each LC-CQD is 248 (calculation details are in the ESI, Scheme S1†). Interestingly, the as-prepared LC-CQDs exhibit strong circular dichroism signals at 245 nm and 350 nm (Fig. 1d), which are significantly different from those of the l-cysteine precursor (Fig. S11†). The developed chiral centers, probably arising from chiral imprint or chiral induction,^21,22^ are preserved in the carbonization process. As is evident from Fig. 1d, LC-CQDs and d-cysteine-rich chiral CQDs (DC-CQDs) display opposing Cotton effects in the range of 200–400 nm, reinforcing the existence of the chirality in both LC-CQDs and DC-CQDs. Thus, it can be inferred that the chirality of the as-prepared LC-CQDs and DC-CQDs is transferred from cysteine to the CQDs.

LC-CQDs display excellent fluorescence properties. As Fig. 1e shows, the normalized UV-FL spectra of the LC-CQDs possess clearly resolved absorption peaks and symmetrical FL peaks, and the blue emission of the LC-CQDs has a maximum wavelength (*λ*_max_) of 420 nm, giving an absolute quantum yield of 68%. The strong fluorescence emission of the LC-CQDs is attributable to the co-doping effect of N and S.^23^ Significantly, the LC-CQDs exhibit superior photostability compared to the FITC dye, fluorescein and the CdTe QDs (regarded as photostable fluorescent labels), as Fig. 1f shows. The fluorescence of FITC, fluorescein and the CdTe QDs was found to be quickly quenched in 30 min under UV irradiation due to severe photobleaching, but of the fluorescence of the LC-CQDs, about 95% of the initial intensity was preserved after 5 h of UV irradiation (Fig. S12†), thus the LC-CQDs were extremely stable in contrast to FITC, fluorescein and the CdTe QDs.

### The Golgi targeting ability of the LC-CQDs

**Fig. 2:**
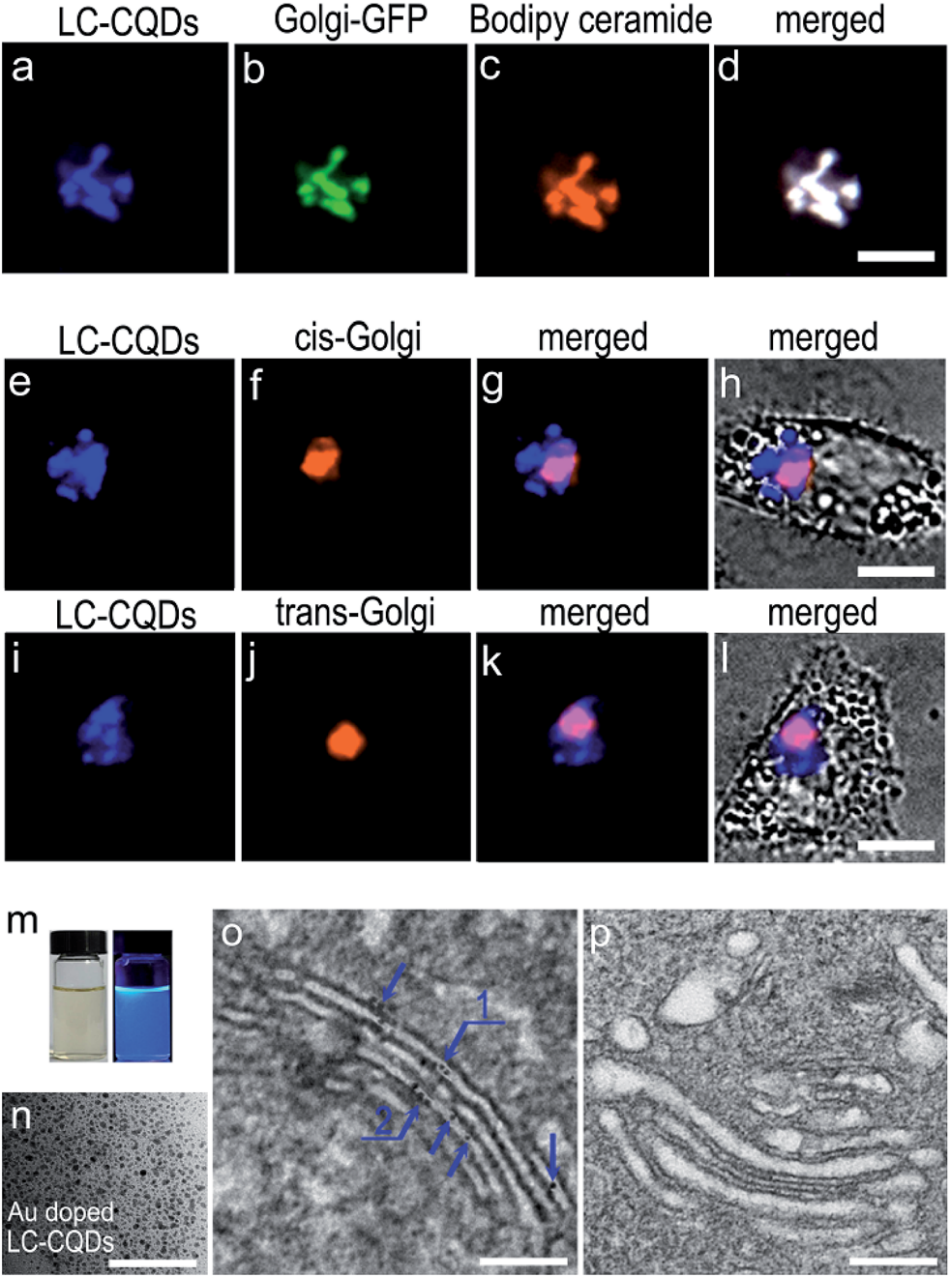
The Golgi targeting ability and the precise location of the LC-CQDs in the Golgi. (a–c) Fluorescence image of the LC-CQDs (blue), Golgi-GFP (green, Golgi specific green fluorescent protein) and Bodipy ceramide (red, Golgi dye) in a HEp-2 cell. (d) Merged image of (a–c). (e) Fluorescence image of the LC-CQDs. (f) Immunofluorescence image of the *cis*-Golgi. The primary antibody was anti-GM130 and the secondary antibody was Rabbit IgG–H&L (Cy3®). (g and h) Merged images of the fluorescence and bright field images. (i) Fluorescence image of the LC-CQDs. (j) Immunofluorescence image of the *trans*-Golgi. The primary antibody was anti-TGN46 and the secondary antibody was Rabbit IgG–H&L (Cy3®). (k and l) Merged images of the fluorescence and bright field images. Scale bar, 10 μm. (m) Photographs of the Au-doped LC-CQDs under illumination by white light (left) and UV (365 nm) light (right). (n) TEM image of the Au-doped LC-CQDs. (o) TEM image of the Golgi after targeting by the LC-CQDs, where the blue arrows indicate the LC-CQDs in the Golgi. (p) TEM image of the Golgi of HEp-2 cells incubated without LC-CQDs; the image shows that no clear black dots were observed in the control Golgi. Scale bar, 200 nm.

The specific Golgi targeting ability of the as-prepared LC-CQDs is demonstrated by co-staining human epithelial cells (HEp-2 cells) with *N*-acetylgalactosaminyltransferase-GFP (Golgi-GFP) and Bodipy ceramide, the two frequently adopted probes for Golgi-staining. Obvious fluorescence in the Golgi is observed (Fig. 2a) after 4 h of incubation of HEp-2 cells with the LC-CQDs (0.5 mg mL^–1^). The fluorescent area matches very well with those of Golgi-GFP and Bodipy ceramide (Fig. 2a–d) with a Pearson’s correlation factor higher than 0.9, indicating that preferential accumulation of the LC-CQDs in the Golgi has occurred (Fig. S13 and S14†). Similar results were also obtained for A549, HepG2, and Hela cells (Fig. S15†). The intracellular uptake and transportation results indicate that the uptake of LC-CQDs is an energy-dependent process mediated by clathrin and caveolae; large quantities of LC-CQDs are transported *via* early endosomes and late endosomes and are eventually transported to the Golgi through the retrograde trafficking route (Fig. S16–S19†). Immunofluorescence results confirm that the LC-CQDs can stain both the *cis*-Golgi and *trans*-Golgi (Fig. 2e–l), and the transmission electron microscopy results show that the LC-CQDs are inside the lumen of the Golgi (Fig. 2m–p).

### Long-term *in situ* Golgi imaging ability of the LC-CQDs

**Fig. 3:**
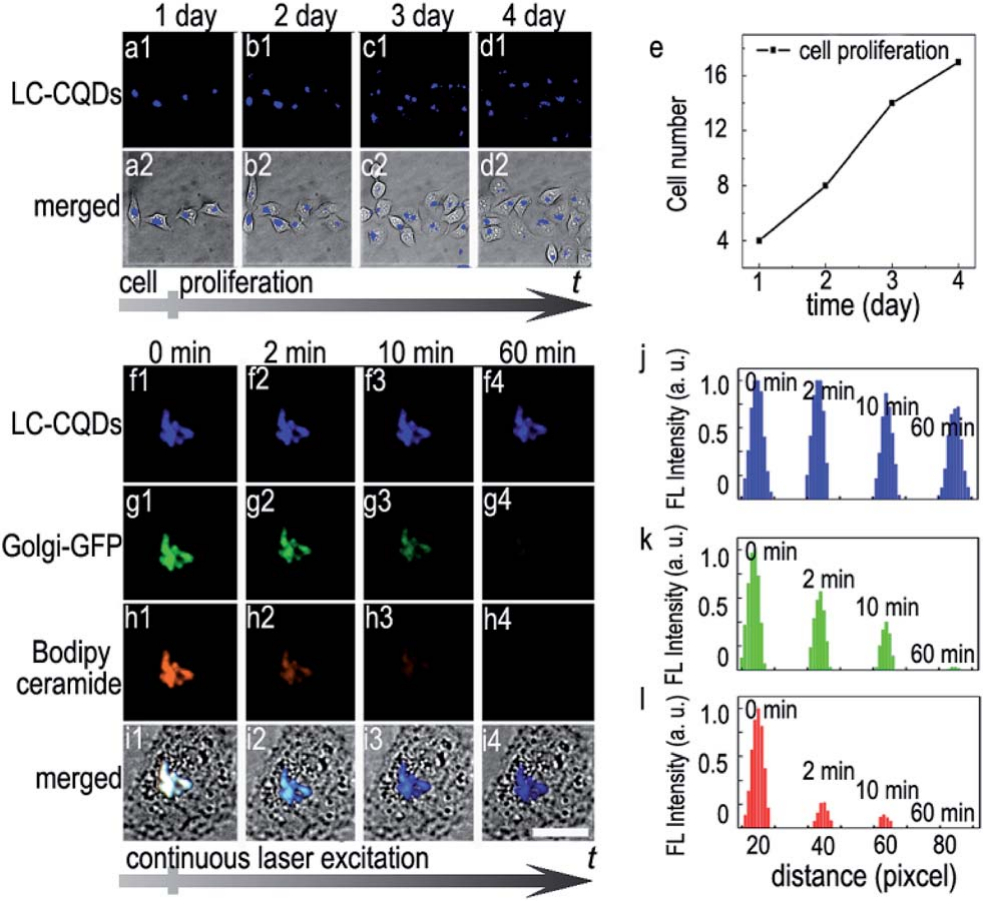
The *in situ* long-term Golgi imaging ability of the LC-CQDs. (a–d) The number of the same group of HEp-2 cells increases from 4 to 17 due to cell proliferation when the Golgi is stained with LC-CQDs. (a1–d1) Fluorescence images of the same group of cells on different days. (a2–d2) Merged images of the fluorescence and bright field images. (e) The plot of cell number with increasing time. (f–i) Photostability of the LC-CQDs (blue), Golgi-GFP (green, Golgi specific green fluorescent protein) and Bodipy ceramide (red, Golgi dye) in cells under continuous laser excitation. Scale bar, 10 μm. (j–l) Changes of the fluorescence intensities of the LC-CQDs (blue), Golgi-GFP (green) and Bodipy ceramide (red) in cells under continuous laser excitation; calculated by Image-Pro Plus 6.0. Scale bar, 20 μm.

To examine the suitability of the LC-CQDs for the long-term *in situ* imaging of the Golgi, different properties, such as imaging duration, biocompatibility and photostability, were evaluated. The cell number increases from four to seventeen, due to cell proliferation (Fig. 3a–e), when HEp-2 cells are incubated with the LC-CQDs for 4 days. In contrast, 1 μM NBD C6-ceramide, whose concentration is considerably lower than that used in typical applications, induced cell rupture and detachment after 2 h of incubation (Fig. S20 and S21†). Furthermore, cell treatment with a typical dose of the LC-CQDs (0.5 mg mL^–1^) does not affect the quantity of secreted proteins, glycoproteins and apoptosis proteins (Fig. S22†), as well as the transportation of semiconductor CdTe quantum dots (QDs) and gold nanoparticles (Fig. S23–S25†), suggesting that LC-CQD labeling does not significantly disturb the function of the Golgi. On the other hand, the fluorescence of the LC-CQDs in the cells remained higher than 80% in the Golgi even under continuous laser excitation for 3600 s (Fig. 3f and j), indicating that the LC-CQDs have good photostability, which is better than those of Bodipy ceramide and Golgi-GFP as the fluorescences of Bodipy ceramide and Golgi-GFP disappear within 600 s (Fig. 3g, h, k and l). The sequential 3D imaging of HEp-2 cells using spinning disk confocal microscopy also demonstrates the superior photostability of the LC-CQDs. For Bodipy ceramide and Golgi-GFP labeling, 70% and 90% loss of fluorescence after 80 3D ‘stacks’ are observed, respectively (Fig. S26b–d†). As a clear contrast, the fluorescence signal of cells treated with the LC-CQDs decreases by only 10% after the same number of 3D ‘stacks’ (Fig. S20a†). Furthermore, the results of the 2′,7′-dichlorofluorescin diacetate (DCF-DA) assay demonstrate that the LC-CQDs would not generate reactive oxygen species during long-term *in situ* Golgi imaging (Fig. S27†). These observations illustrate that the LC-CQDs are feasible for the *in situ* long-term fluorescence observation of the Golgi.

### The Golgi targeting mechanism of the LC-CQDs

Both the l-type stereo configuration and the existence of free thiol groups are proven to be necessary for targeting the Golgi apparatus. Compared with LC-CQDs, DC-CQDs prepared from d-cysteine and citric acid (Fig. 1d and S28–S30†) exhibit a limited Golgi targeting ability (Fig. S31a†) with a Pearson’s correlation factor of 0.35, indicating that the chirality of the CQDs strongly influences the targeting ability towards the Golgi. CQDs prepared from precursors without chiral centers, *e.g.* thioacetamide, are found to randomly spread in cells with a Pearson’s correlation factor of 0.28 (Fig. S31e and S32–S34†), reinforcing the significance of a proper stereo structure of the CQDs for Golgi targeting. Furthermore, the existence of free thiol groups is proven to be essential for Golgi targeting. Carbon dots prepared from precursors carrying no free thiol groups, *S*-methyl-l-cysteine (Fig. S35–S37†) or l-alanine (Fig. S38–S40†), are found to have very low Golgi targeting abilities as indicated by Pearson’s correlation factors of 0.21 and 0.17 (Fig. S31c and d†), respectively. A further competitive inhibition experiment shows that molecules without thiol groups cannot inhibit the targeting ability of the LC-CQDs or compete with the LC-CQDs in binding to the Golgi as l-cysteine does (Fig. S41†). These observations solidify the necessity of free thiol groups for the Golgi targeting and also suggest that the LC-CQDs may bind to the sulfhydryl receptor site of the Golgi through the formation of disulphide bonds in the oxidizing environment of the Golgi lumen.^24^

### l-Cysteine induced Golgi targeting

**Fig. 4:**
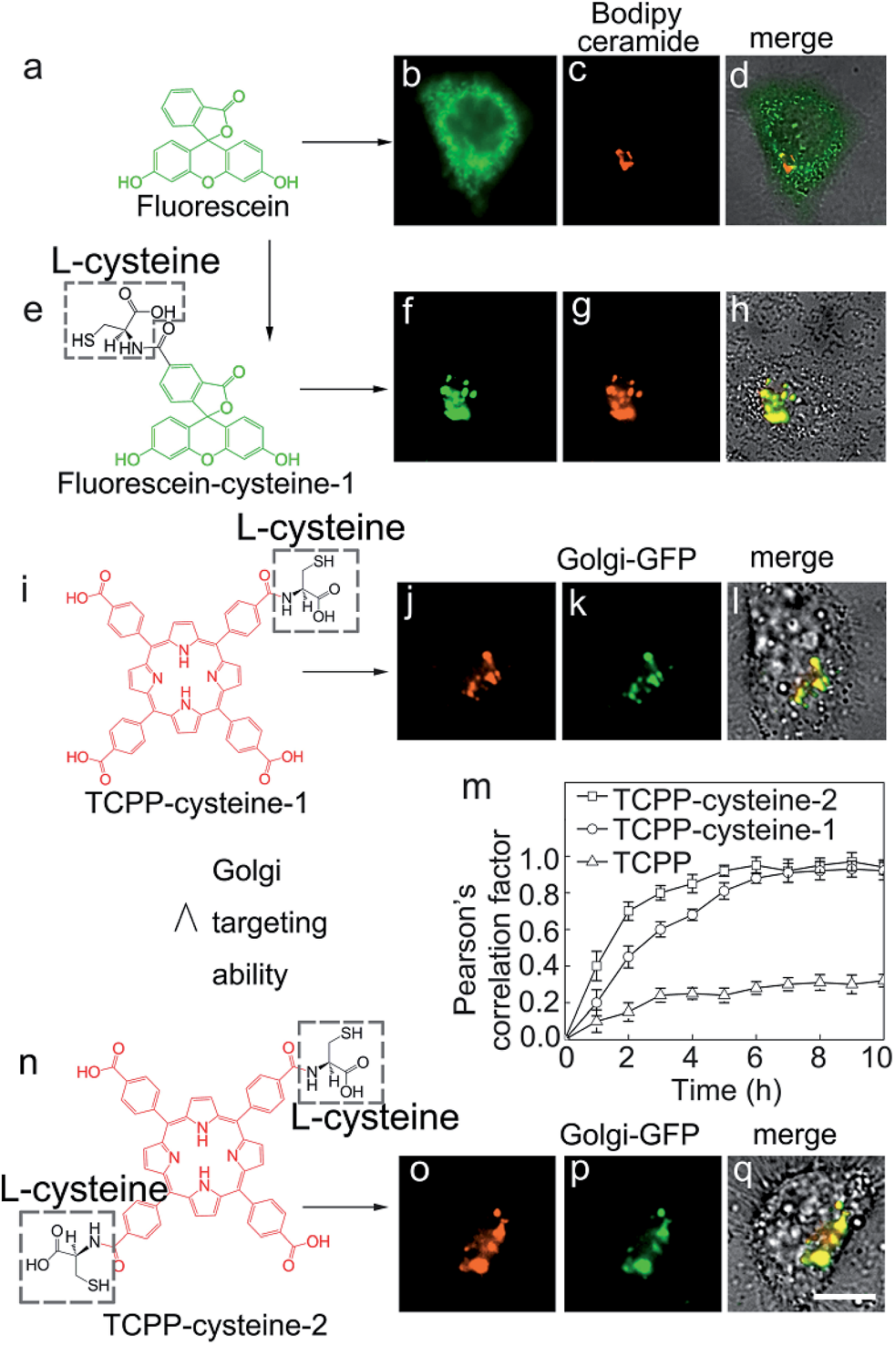
The Golgi targeting ability of l-cysteine functionalized molecules. (a) Chemical structure of fluorescein. (b) Fluorescence image of fluorescein in an HEp-2 cell. (c) Fluorescence image of Bodipy ceramide (red, Golgi specific dye). (d) Merged image of fluorescein, Bodipy ceramide and the cell. (e) Chemical structure of fluorescein–cysteine-1. (f) Fluorescence image of fluorescein. (g) Fluorescence image of Bodipy ceramide (red, Golgi specific dye). (h) Merged image of fluorescein–cysteine-1, Bodipy ceramide and the cell. (i) Chemical structure of TCPP–cysteine-1. (j) Fluorescence image of TCPP–cysteine-1. (k) Fluorescence image of GalNAcT-GFP (Golgi-GFP, Golgi specific green fluorescent protein). (l) Merged image of TCPP–cysteine-1, GalNAcT-GFP and the cell. (m) Pearson’s correlation factor between TCPP–cysteine-2/TCPP–cysteine-1/TCPP and GalNAcT-GFP at different times. Calculated using Image-Pro Plus 6.0. The error bars represent the standard deviations from the five replicate experiments. (n) Chemical structure of TCPP–cysteine-2. (o) Fluorescence image of TCPP–cysteine-1. (p) Fluorescence image of GalNAcT-GFP. (q) Merged image of TCPP–cysteine-2, GalNAcT GFP and the cell. Scale bar, 10 μm.

**Fig. 5:**
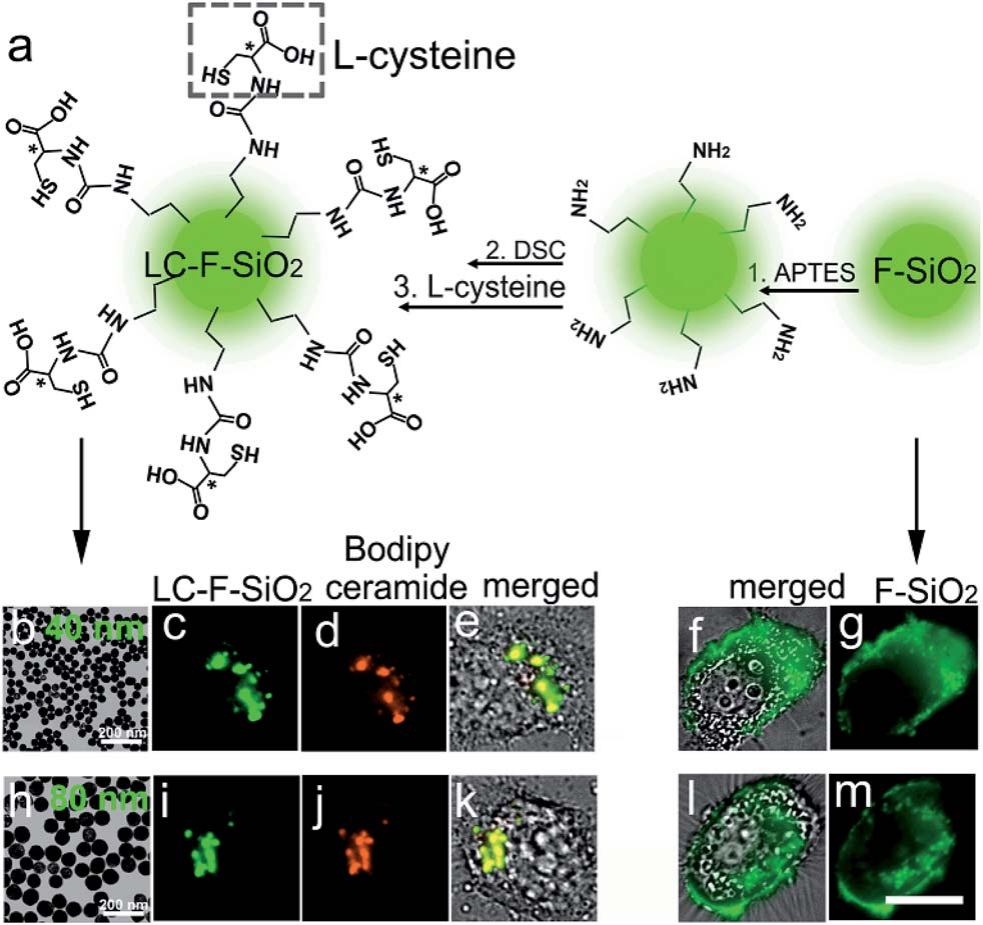
The Golgi targeting ability of l-cysteine functionalized silica nanoparticles. (a) The synthesis process of the l-cysteine functionalized silica nanoparticles. (b) Transmission electron microscopy (TEM) image of the 40 nm l-cysteine functionalized fluorescent silica nanoparticles (LC-F-SiO_2_). (c) Fluorescence image of the 40 nm LC-F-SiO_2_ in an HEp-2 cell. (d) Fluorescence image of Bodipy ceramide (red, Golgi specific dye). (e) Merged image of the 40 nm LC-F-SiO_2_, Bodipy ceramide and the cell. (f) Merged image of the 40 nm fluorescent silica nanoparticles (F-SiO_2_) in an HEp-2 cell. (g) Fluorescent image of the 40 nm F-SiO_2_ in the HEp-2 cell. (h) Transmission electron microscopy (TEM) image of the 80 nm l-cysteine functionalized fluorescent silica nanoparticles (LC-F-SiO_2_). (i) Fluorescence image of the 80 nm LC-F-SiO_2_ in an HEp-2 cell. (j) Fluorescence image of Bodipy ceramide. (k) Merged image of the 80 nm LFF-SiO_2_, Bodipy ceramide and the cell. (l) Merged image of the 80 nm fluorescent silica nanoparticles (F-SiO_2_) in the HEp-2 cell. (m) Fluorescence image of 80 nm F-SiO_2_ in the HEp-2 cell. Scale bar, 10 μm.

In order to demonstrate the feasibility of l-cysteine in Golgi-targeting, we link l-cysteine with different materials using chemical approaches. As Fig. 4a shows, HEp-2 cells are incubated with l-cysteine functionalized fluorescein and a complete colocalization is observed for the fluorescein–cysteine molecules and Bodipy ceramide with a Person’s correlation factor greater than 0.94 (Fig. 4b–d and S42–S51†). In contrast, fluorescein without cysteine functionalization is found to readily spread over the whole cells (Fig. 4e–h). Similar results are observed for l-cysteine modified silica nanoparticles with sizes of 40 nm and 80 nm (Fig. 5), further strengthening the hypothesis that l-cysteine could target the Golgi. In order to identify the multivalent effect of l-cysteine, *meso*-tetra(4-carboxyphenyl)porphine (TCPP) is linked with different numbers of l-cysteine residues. Unfunctionalized TCPP is found to spread over the whole cells, while single cysteine functionalized TCPP colocalizes well with Golgi-GFP (Fig. 4i–m) after 5 h incubation. Double l-cysteine functionalized TCPP needs only 3 h to match with Golgi-GFP (Fig. 4m and n and S52–S58†), suggesting that the Golgi targeting ability is closely associated with the number of l-cysteine residues and the multivalent effect of l-cysteine can improve the Golgi targeting ability. Similarly, the Golgi targeting ability of the LC-CQDs would improve with an increasing number of cysteine residues on their surface (Fig. S59†). The multivalent effect of the LC-CQDs is also verified by the fluorescence recovery after photobleaching (FRAP) (Fig. S60 and S61†). These results confirm that l-cysteine is an effective agent for specific Golgi targeting.

### Viral infection induced Golgi fragmentation

**Fig. 6:**
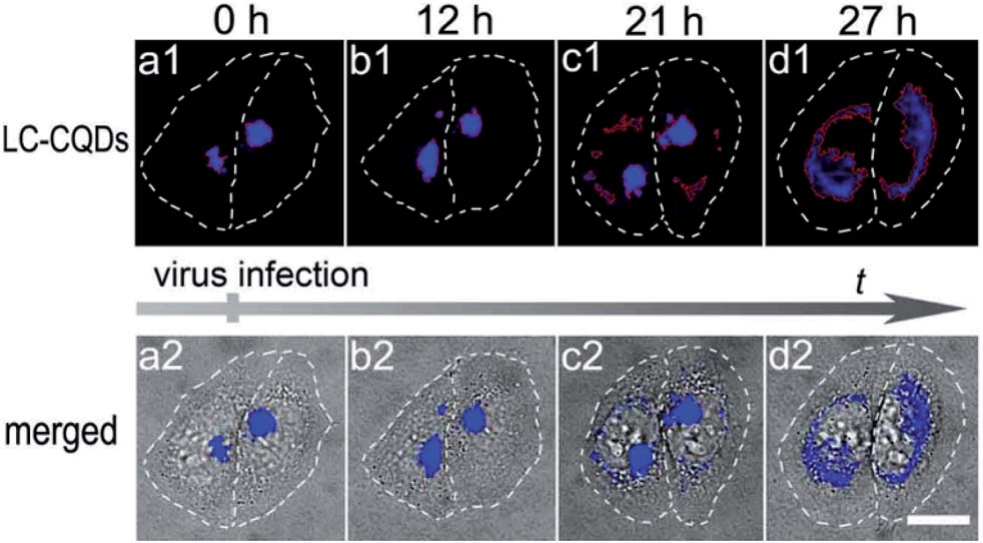
Morphological changes in the Golgi apparatus of HEp-2 cells during viral infection. (a1–d1) Fluorescence images of the Golgi stained with the LC-CQDs. (a2–d2) Merged images of the fluorescence and bright field images. Scale bar, 20 μm.

The long-term *in situ* targeting ability of the LC-CQDs could be further demonstrated by visualizing the dynamic morphology changes of the Golgi at the single-cell level during viral infection. Stained with the LC-CQDs, the Golgi apparatus of HEp-2 cells emits blue fluorescence (Fig. 6a). After the infection of HEp-2 cells with respiratory syncytial virus (RSV) for 12 h, the Golgi breaks into fragments and becomes scattered (Fig. 6b). The collapse becomes even more serious (Fig. 6c) after 21 h. The Golgi morphology changes might be a more sensitive indicator of cell vital state changes, because the cell morphology does not show an obvious change during the Golgi fragmentation (Fig. 6a–d). Similar morphological changes were also obtained for A549, HepG2, and Hela cells during the process of viral infection (Fig. S62†). Identification of the viral attack-induced Golgi response reveals a transition stage from the living state to the death of the cells, thus providing a new and sensitive mode of investigating diagnostically relevant changes in the early stages of viral infection. To the best of our knowledge, this is the first report about the Golgi response in the early stages of viral infection.

## Conclusions

We have successfully demonstrated an approach to prepare chiral carbon quantum dots (l-type stereo structure, LC-CQDs) with abundant cysteine residues. The LC-CQDs exhibit unique properties, including a high quantum yield (68%), high photo-stability, as well as favorable biocompatibility. More importantly, the LC-CQDs exhibit a distinctive capacity for long-term *in situ* Golgi imaging *via* the l-type stereo structure and free thiol groups. Due to their long-term *in situ* Golgi imaging ability, the LC-CQDs are able to trace the real time process of intracellular injury, such as viral infection, by monitoring the morphological changes of the Golgi apparatus. Thus a transition stage from the living state to the death of the cells during the process of viral infection has been observed, providing an innovative mode of investigating diagnostically relevant changes in the early stages of viral infection. Furthermore, the exploration of cysteine for Golgi targeting provides an innovative subcellular targeting strategy for drug design and delivery, and thus illustrating that functional cellular CQDs that can target subcellular organelles such as Golgi body show high promise in the explorations of cellular activities of typical subcellular organelles.

## Experimental

### Synthesis of the LC-CQDs

The LC-CQDs were prepared by directly pyrolysing l-cysteine and citric acid (CA). CA monohydrate (2 g, 9.5 mmol) was at first placed in a 50 mL flask and heated at 200 °C for 20 min, and then l-cysteine (1.5 g, 12.4 mmol) was added. After stirring with a glass rod, the yellow mixture was kept at 200 °C for 60 min until a dark product was obtained. 2 mL of ultra-pure water was added to the mix of carbon quantum dots with cysteine and heating was continued if the yellow mixture did not turn dark. The dark product was finally neutralized with KOH solution (10 mL, 1.3 mol L^–1^). The LC-CQDs were collected by removing the large particles through filtering using 0.22 μm membranes, and then dialyzing against ultra-pure water for 48 h to remove small organic molecules and salts (3.5k MWCO, Spectra/Pro 3 dialysis sack). Other carbon quantum dots were synthesized by replacing the l-cysteine with d-cysteine, l-alanine, *S*-methyl-l-cysteine, thioacetamide and a gold–cysteine complex (1.5 g cysteine, 5 mL 5% HAuCl_4_).

### Synthesis of l-cysteine functionalized silica nanoparticles

Methanol (10 mL), ultra-pure water (3 mL), and concentrated ammonia (0.7 mL) were mixed at 30 °C under stirring. Tetraethyl orthosilicate (200 μL) was then added to the solvent mixture followed by the addition of a fluorescent complex (20 μL methanol, 0.16 mg fluorescein isothiocyanate (FITC), and 4.8 μL 3-aminopropyltriethoxysilane (APTES). The fluorescent complex solution should be mixed and stored at 4 °C for one day before the synthesis process). After 4 h, the products were collected by centrifugation at 12k rpm and washed with ethanol. By adjusting the amount of concentrated ammonia used under the same conditions, the particle sizes of the silica nanoparticles were tuned to 40 nm (concentrated ammonia, 1.2 mL), and 80 nm (concentrated ammonia, 0.7 mL). Subsequently, the as-prepared silica nanoparticles were dispersed in 15 mL of ethanol followed by the addition of 200 μL APTES. After 12 h, the amine-functionalized silica nanoparticles were collected and washed with ethanol. The amine-functionalized silica nanoparticles were then redispersed in 15 mL acetone followed by the addition of *N*,*N*′-disuccinimidyl carbonate (DSC, 300 mg) and *N*,*N*-diisopropylethylamine (DIEA, 200 μL). After another 12 h, the DSC-functionalized silica nanoparticles were collected and washed with acetone. Finally, the DSC-functionalized silica nanoparticles were redispersed in 8 mL l-cysteine (Sigma-Aldrich) complex solution (8 mL ultra-pure water, 300 mg l-cysteine and 400 μL DIEA) for 4 h. The l-cysteine functionalized fluorescent silica nanoparticles (LC-F-SiO_2_) were collected by centrifugation at 12k rpm and washed with ultra-pure water. Some large products could be removed by centrifugation at 4k rpm.

### Cell imaging

In the cellular experiments, cells were cultured in RPMI 1640 (Hyclone) media supplemented with 10% foetal bovine serum (FBS, Hyclone), 100 U mL^–1^ penicillin G and 100 μg mL^–1^ streptomycin sulphate in a CO_2_ incubator at 37 °C. One day prior to adding the LC-CQDs, the cells were seeded on 35 mm glass-bottom dishes (NEST. Corp.). The cells were then incubated with the LC-CQDs (0.5 mg mL^–1^, diluted by RPMI 1640 supplemented with 2% FBS) overnight. After washing three times with phosphate buffered saline (PBS), the cells were observed under a confocal microscope. Fluorescein, fluorescein–cysteine-1, fluorescein–cysteine-2, TCPP, TCPP–cysteine-1, TCPP–cysteine-2, F-SiO_2_ and LC-F-SiO_2_ were incubated and observed under the same conditions. Fluorescein–cysteine-1, fluorescein–cysteine-2, TCPP, TCPP–cysteine-1, and TCPP–cysteine-2 were designed by our group and synthesized by Sangon Biotech (Shanghai) Co., Ltd.

Bodipy TR C_5_-ceramide (Thermo Fisher Scientific) or NBD C_6_-ceramide (Thermo Fisher Scientific) can be administered to cells as a complex with bovine serum albumin (BSA). The cells were washed until they were free of culture media and incubated with Bodipy TR C_5_-ceramide (5 μM) for 30 min at 4 °C. The cells were then washed, incubated in 2% BSA solution for 90 min at 25 °C, and finally observed under a fluorescence microscope. CellLight® Golgi-GFP (Thermo Fisher Scientific) was added to the cells, incubated overnight, and the cells were ready to image in the morning.

All the carbon quantum dots were excited at 360–370 nm or 405 nm and detected with a barrier filter BA 420–460 nm. Golgi-GFP, NBD C_6_-ceramide, F-SiO_2_, LC-F-SiO_2_, fluorescein, fluorescein–cysteine-1, and fluorescein–cysteine-2 were excited at 470–490 nm and detected with a barrier filter BA 510–550 nm. BODIPY TR C_5_-ceramide, TCPP, TCPP–cysteine-1 and TCPP–cysteine-2 were excited at 530–550 nm and detected with a barrier filter BA 575–625 nm.

The HEp-2 cells used in this experiment are the laryngeal cancer cell lines. The respiratory syncytial virus (RSV) used in this experiment is effective at infecting HEp-2 cells. In order to monitor the morphology changes of the Golgi during the viral infection, we selected Hep-2 cells as the host cells.

The data in the main text and ESI† were obtained from replicate experiments (*n* = 5).

### Electronic microscopy study of the LC-CQDs in the Golgi

Cells grown in cell culture flasks were incubated with 1 mg mL^–1^ Au-doped LC-CQDs for 48 h, washed, and fixed in 1% glutaraldehyde. The cells were then washed with 0.1 M PBS buffer and treated with 0.5% OsO_4_ for 1 h at 4 °C. After rinsing with ultra-pure water, the cells were stained with saturated uranyl acetate in 70% ethanol for 24 h at 25 °C. The cells were then dehydrated in ethanol and embedded in Epon. Thin sections were obtained with an ultramicrotome. Electron micrographs were obtained with an electron microscope (Transmission Electron Microscope Hitachi-7500). Control samples were treated as described above, except that they were not incubated with LC-CQDs.

### Immunofluorescence study of the LC-CQDs in the Golgi

The cells were fixed in 4% paraformaldehyde for 20 min at 25 °C and permeabilized with 0.1% Triton X 100 for 2 min at 25 °C. The primary antibodies were diluted 1/200 and incubated with samples for 1 h at 37 °C. The secondary antibody was the goat polyclonal secondary antibody to Rabbit IgG–H&L (Cy3®) used at a 1/500 dilution for 1 h. 2% BSA was used for the blocking steps. All antibodies were purchased from Abcam.

### Viral infection

Virus samples were diluted with RPMI 1640 and then introduced to the cells. The Golgi apparatus of the cells had been labelled by LC-CQDs before the addition of the virus. After incubating with RSV for 2 h at 37 °C, the infected cells were cultured in RPMI 1640 supplemented with LC-CQDs and 2% FBS in a 5% CO_2_ incubator. The morphology of the Golgi apparatus of the same cell was observed at different times.

HEp-2 cells used in this experiment were the laryngeal cancer cell lines. The respiratory syncytial virus (RSV) used in this experiment was effective at infecting HEp-2 cells.

### Quantum yield

The absolute photoluminescence quantum yield was measured using Quantaurus-QY (Hamamatsu, Japan).

## References

[cit1] Lipsky N., Pagano R. (1985). Science.

[cit2] Rajendran L., Knolker H.-J., Simons K. (2010). Nat. Rev. Drug Discovery.

[cit3] Marsh B. J., Howell K. E. (2002). Nat. Rev. Mol. Cell Biol..

[cit4] Bykovskaja S. N., Rytenko A. N., Rauschenbach M. O., Bykovsky A. F. (1978). Cell. Immunol..

[cit5] Lipsky N. G., Pagano R. E. (1983). Proc. Natl. Acad. Sci. U. S. A..

[cit6] Erdmann R. S., Takakura H., Thompson A. D., Rivera-Molina F., Allgeyer E. S., Bewersdorf J., Toomre D., Schepartz A. (2014). Angew. Chem., Int. Ed..

[cit7] Louvard D., Reggio H., Warren G. (1982). J. Cell Biol..

[cit8] Virtanen I., Ekblom P., Laurila P. (1980). J. Cell Biol..

[cit9] Xu X., Ray R., Gu Y., Ploehn H. J., Gearheart L., Raker K., Scrivens W. A. (2004). J. Am. Chem. Soc..

[cit10] Yuan Y. H., Li R. S., Wang Q., Wu Z. L., Wang J., Liu H., Huang C. Z. (2015). Nanoscale.

[cit11] Kalytchuk S., Poláková K., Wang Y., Froning J. P., Cepe K., Rogach A. L., Zbořil R. (2017). ACS Nano.

[cit12] Cayuela A., Kennedy S. R., Soriano M. L., Jones C. D., Valcarcel M., Steed J. W. (2015). Chem. Sci..

[cit13] Mu Y., Wang N., Sun Z., Wang J., Li J., Yu J. (2016). Chem. Sci..

[cit14] Liu H., Ye T., Mao C. (2007). Angew. Chem., Int. Ed..

[cit15] Zheng X. T., Ananthanarayanan A., Luo K. Q., Chen P. (2015). Small.

[cit16] Ding C., Zhu A., Tian Y. (2014). Acc. Chem. Res..

[cit17] Trachtenberg J. T., Chen B. E., Knott G. W., Feng G., Sanes J. R., Welker E., Svoboda K. (2002). Nature.

[cit18] Maeda Y., Beznoussenko G. V., Lint J. V., Mironov A. A., Malhotra V. (2001). EMBO J..

[cit19] Aoki D., Lee N., Yamaguchi N., Dubois C., Fukuda M. N. (1992). Proc. Natl. Acad. Sci. U. S. A..

[cit20] Yang J., Zhang Y., Gautam S., Liu L., Dey J., Chen W., Mason R. P., Serrano C. A., Schug K. A., Tang L. (2009). Proc. Natl. Acad. Sci. U. S. A..

[cit21] Morris R. E., Bu X. (2010). Nat. Chem..

[cit22] Wattanakit C., Côme Y. B. S., Lapeyre V., Bopp P. A., Heim M., Yadnum S., Nokbin S., Warakulwit C., Limtrakul J., Kuhn A. (2014). Nat. Commun..

[cit23] Dong Y., Pang H., Yang H. B., Guo C., Shao J., Chi Y., Li C. M., Yu T. (2013). Angew. Chem., Int. Ed..

[cit24] Maeda K., Hägglund P., Finnie C., Svensson B., Henriksen A. (2006). Structure.

