# Supplementary material for: Chiral nanoprobes for targeting and long-term imaging of the Golgi apparatus
Source: Chem Sci. 2017 Jul 13;8(10):6829–35. doi: 10.1039/c7sc01316g (PMC5643954; doi:10.1039/c7sc01316g)
Supplement: Supplementary file 1 [file SC-008-C7SC01316G-s001.pdf]

Electronic Supplementary Material (ESI) for Chemical Science.
This journal is © The Royal Society of Chemistry 2017

Electronic Supplementary Information (ESI) for:

Chiral nanoprobes for targeting and long-term imaging of the Golgi apparatus

Rong Sheng Li,[a] *Peng Fei Gao,*[a] *Hong Zhi Zhang,*[a] *Lin Ling Zheng,*[a] *Chun Mei Li,*[a] *Jian Wang,*[a,*] *Yuan Fang Li,*[b] *Feng Liu,*[c] *Na Li,*[c,*] *and Cheng Zhi Huang*[a,b,*]

[a] Key Laboratory of Luminescent and Real-Time Analytical Chemistry (Southwest University), Ministry of Education, College of Pharmaceutical Sciences, Southwest University, Chongqing 400716, China.

[b] College of Chemistry and Chemical Engineering, Southwest University, Chongqing 400715, China.

[c] Beijing National Laboratory for Molecular Sciences (BNLMS), Key Laboratory of Bioorganic Chemistry and Molecular Engineering of Ministry of Education, Institute of Analytical Chemistry, College of Chemistry and Molecular Engineering, Peking University, Beijing 100871, China

*Correspondence and requests for materials should be addressed to W, J. L, N or H, C. (email: chengzhi@swu.edu.cn, wj123456@swu.edu.cn, or lina@pku.edu.cn).

Materials and Apparatus. Fluorescein-cysteine-1, Fluorescein-cysteine-2, TCPP-cysteine-1, and TCPP-cysteine-2 were designed by our group and synthesized by Sangon Biotech (Shanghai) Co., Ltd. All other chemicals were of analytical reagent and purchased from Sigma-Aldrich. High-resolution transmission electron microscopy (HRTEM) imaging was recorded with Tecnai G2 F20 S-TWIN microscopy (FEI, USA). An ESCALAB 250 X-ray photoelectron spectrometer (thermo, USA) was used for recording XPS spectra. A FTIR-4800 Fourier transform infrared (FT-IR) spectrophotometer (Shimadzu, Japan) was used to record the IR spectra. Absorption spectra were scanned with an UV-3600 spectrophotometer (Shimadzu, Japan). Photoluminescence (PL) spectra were recorded with an F-2500 fluorospectrophotometer (Hitachi, Japan). PL lifetimes were measured using a Fluorolog-3 fluorescence spectrometer (Horiba Jobin Yvon Inc., France). Fluorescent images were acquired using an Olympus IX-81 inverted microscope equipped with an Olympus IX2-DSU confocal scanning system and a Rolera-MGi EMCCD.

The calculation of cysteine residues on the surface of LC-CQDs. We investigated the number of L-cysteine molecules on each carbon quantum dots by calculating the whole number of L-cysteine on the surface of LC-CQDs and the average molar mass of LC-CQDs.

Ellman's reagent (5,5'-dithiobis-(2-nitrobenzoic acid); DTNB) was used to quantify the number of thiol groups on the surface of LC-CQDs. As shown in Scheme S1, thiols groups react with DTNB, cleaving the disulfide bond to give 2-nitro-5-thiobenzoate (TNB^{-}), which ionizes to the TNB^{2-} dianion in water at alkaline pH. This TNB^{2-} ion has a yellow color. The reaction between LC-CQDs and DTNB is rapid and stoichiometric, with the addition of one mole of thiol releasing one mole of TNB^{2-}. The TNB^{2-} is quantified in a spectrophotometer by measuring the absorbance of visible light at 412 nm. The calibration curve of optical density (OD) is plotted as function of standard cysteine concentrations. We get the number of cysteine on the surface of LC-CQDs according to the absorbance of TNB^{2-} at 412 nm.

The average molar mass of LC-CQDs is 346 kD, which is calculated according to their diameter (~8.5 nm), topographic heights (~18 layers of graphene-like sheets), and lattice spacing (0.21 nm, in-plane lattice spacing of graphene (100)).

Synthetic method. Our method is generalizable for incorporating other groups on the surface of carbon quantum dots. The carbon quantum dots used in this work are prepared by pyrolizing citric acid and they have a lot of carboxyl groups on the surface. These carboxyl groups could bind with amino groups or hydroxy groups by a dehydration-condensation reaction. The water produced in the reaction could be evaporated from the reaction substances when the reaction temperature is higher than 100 ^{o}C (the temperature in this work is 200 ^{o}C). The dehydration-condensation reaction preceeds by the evaporation of water produced in the reaction. Molecules which have animo groups or hydroxy groups could bind on the surface of carbon quantum dots using this method. It is need to note that the temperature should not be higher than the decomposition temperature of incorporating molecules.

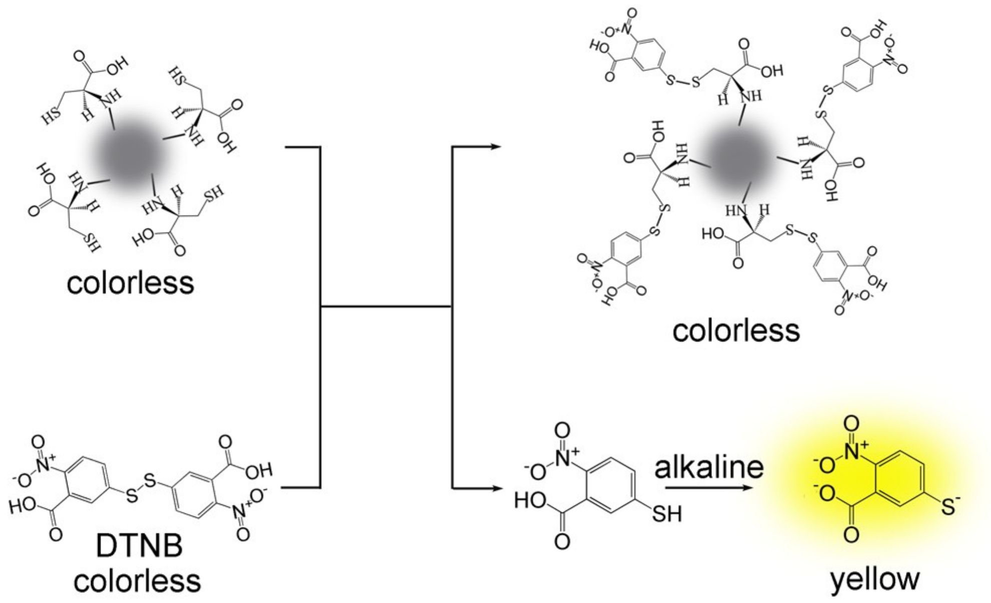

Scheme S1. Schematic illustration of the reaction between LC-CQDs and DTNB.

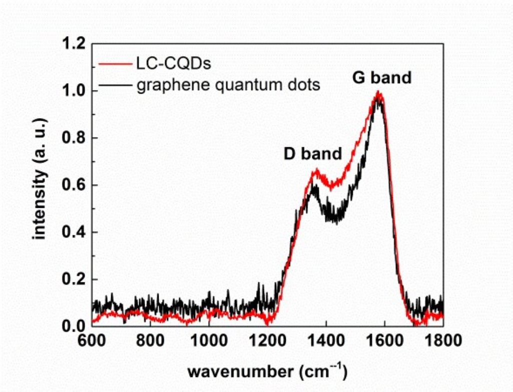

Figure S1. Raman spectra of LC-CQDs and graphene quantum dots (the cores of LC-CQDs). D band was caused by the *sp*^{3}-hybrized carbon atoms, while G band was caused by the *sp*^{2}-hybrized carbon atoms, indicating that the as-prepared LC-CQDs have core-shell structure, wherein the core is composed by the graphene layer with the *sp*^{2}-hybrized carbon atoms and the shell is composed by the functional groups containing sp3-hybrized carbon atoms.

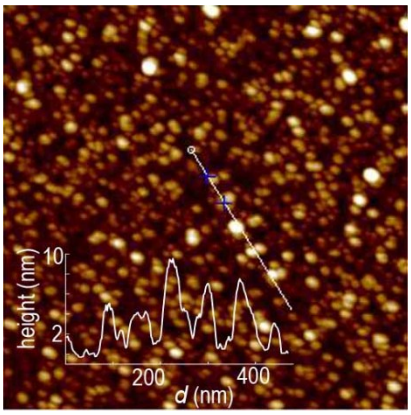

Figure S2. Atomic force microscope (AFM) image of LC-CQDs. Their topographic heights were from 3.5 to 9.6 nm, showing that LC-CQDs consisted primarily of eleven to thirty layers of graphene-like sheets.

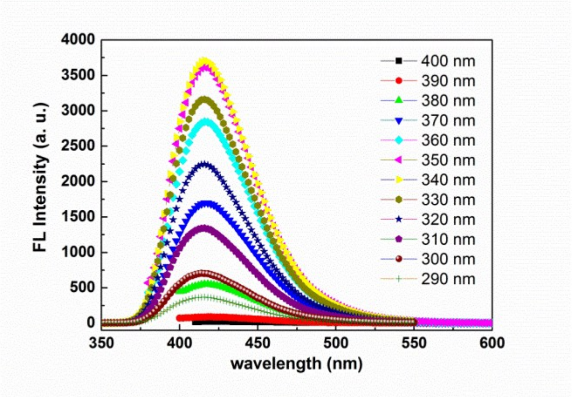

Figure S3. Fluorescence spectra of the aqueous solution of LC-CQDs with excitation at different wavelengths, indicating that the emission of LC-CQDs is independent of the excitation wavelength.

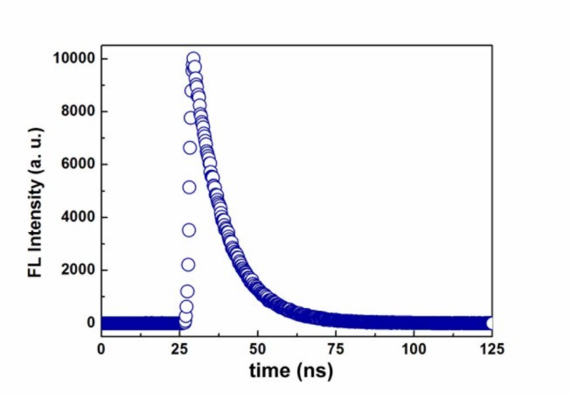

Figure S4. Fluorescence decays of LC-CQDs (l_{ex}=350 nm, l_{em}=420 nm). The fluorescence life time of LC-CQDs is 10.07 ns.

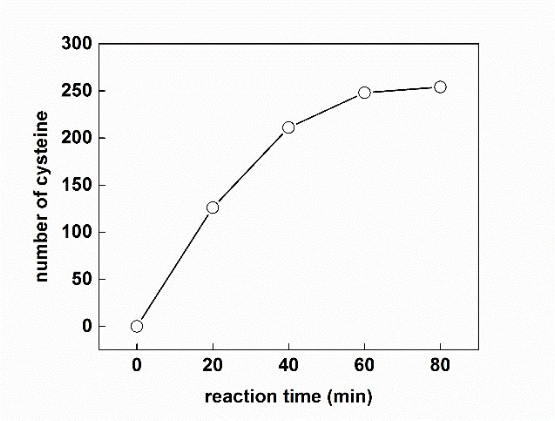

Figure S5. The relationship between the reaction time and the number of cysteine on the surface of LC-CQDs.

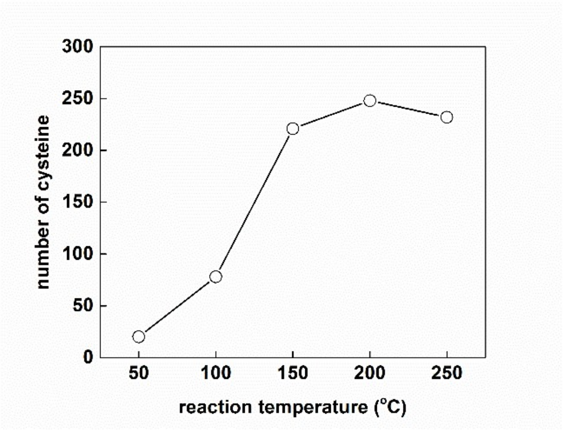

Figure S6. The relationship between the reaction temperature and the number of cysteine on the surface of LC-CQDs.

Note: We controlled the number of cysteine on the carbon quantum dots by adjusting the reaction time and temperature. As shown in Figure S5 and S6, the number would increase as the increasing of the reaction time (0−60 min) or temperature (0−200 °C).

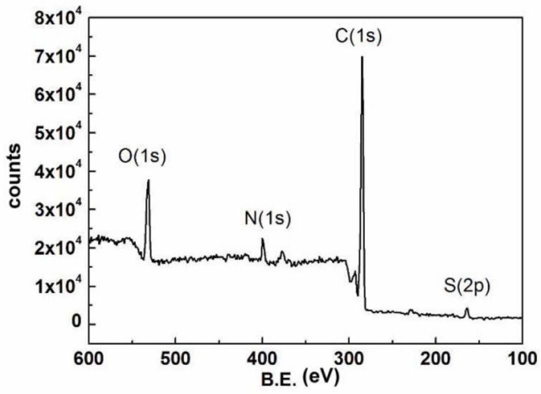

Figure S7. XPS spectra of LC-CQDs, indicating that the as-prepared LC-CQDs containing C, N, O, and S.

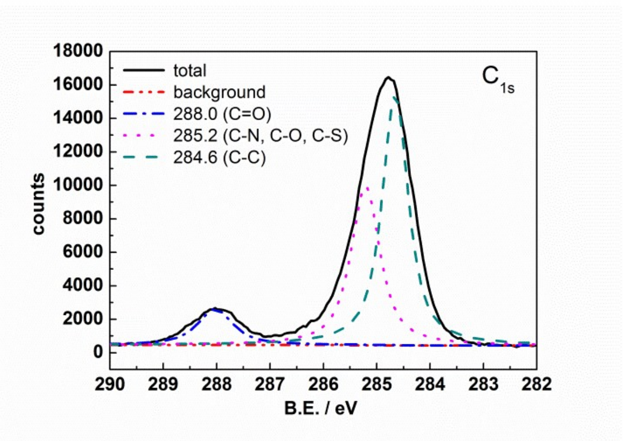

Figure S8. C_{1s} peaks of high-resolution XPS spectra of LC-CQDs, indicating carbon atoms of LC-CQDs bond with C, N, O, and S.

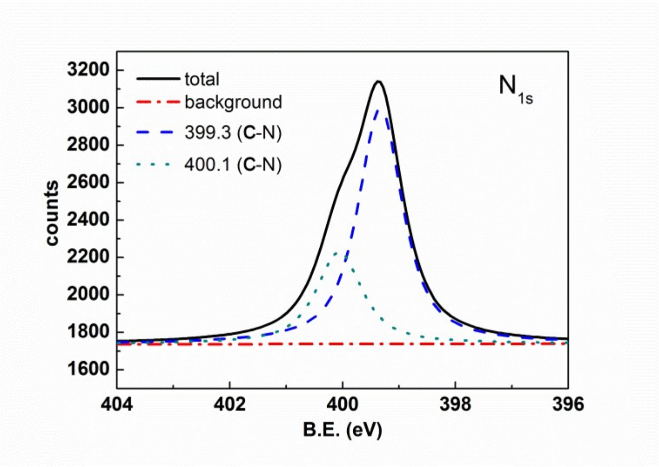

Figure S9. N_{1s} peaks of high-resolution XPS spectra, indicating the chemical bonds between carbon and nitrogen.

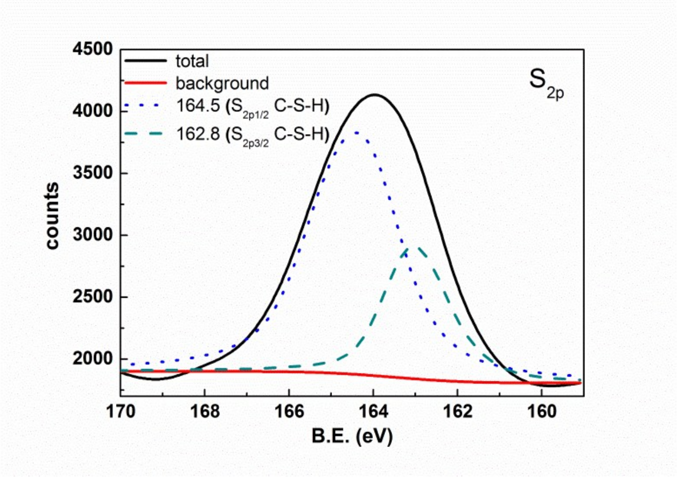

Figure S10. S_{2p} peaks of high-resolution XPS spectra of LC-CQDs, indicating the chemical bonds between carbon and sulfur.

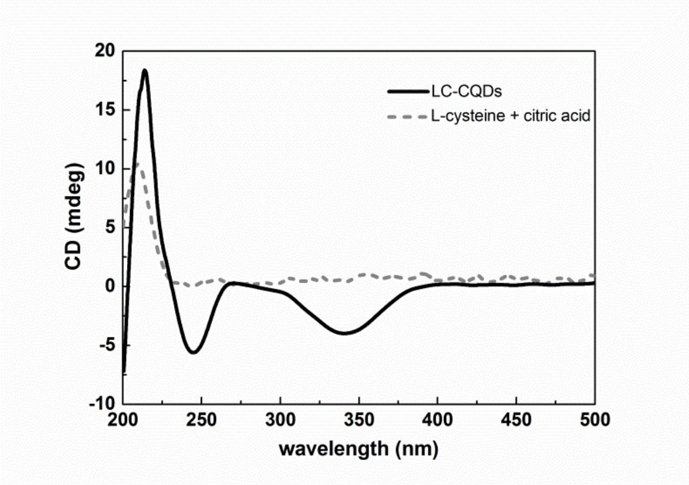

Figure S11. Circular dichroism (CD) spectra of LC-CQDs (solid curve) and their precursors (L-cysteine and citric acid, dot curve). Notes: the circular dichroism peaks of LC-CQDs at 245 nm and 350 nm indicate the formation of new chiral center. The CD peak of LC-CQDs in 220 nm is similar to the CD peak of L-cysteine, indicating that the chiral center of L-cysteine has been inherited by LC-CQDs.

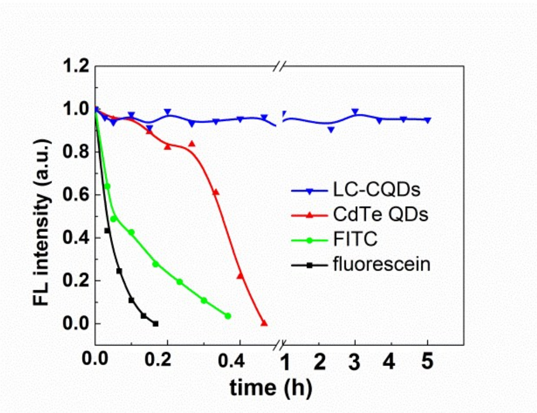

Figure S12. (*A*) Photostability comparison of FITC, fluorescence, CdTe CQDs and LC-CQDs. (*B*–*F*) photographs of FITC, fluorescein, CdTe QDs, and LC-CQDs aqueous solution under UV irradiation for different time. All samples are continuously irradiated by a 280 W xenon lamp. The results indicate the excellent photostability of LC-CQDs, which is better than CdTe quantum dots, FITC, and fluorescein.

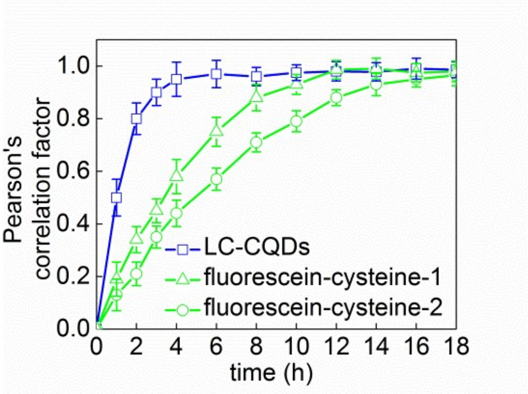

Figure S13. Pearson's correlation factor between LC-CQDs (blue) and Golgi (Golgi-GFP, green) or between fluorescence-cysteine dyes (green) and Golgi (Bodipy ceramide, red) in different time, calculated by Image-Pro Plus 6.0. Error bars represent standard deviations from five replicate experiments.

Note: Fluorescein-cysteine complexes require over 10 h for the Golgi imaging. Considering that only one cysteine was linked with a fluorescein dye molecule, we suppose that the longer targeting time to the Golgi for those dyes (10 h) than that for LC-CQDs (4 h) may be attributed to the number of cysteine molecules. Yet, a large number of cysteine molecules were retained on the surface of L-CQDs, greatly enhancing the Golgi affinity of L-CQDs owing to the multivalent attachment effects.

Cellular Uptake and Intracellular Transportation of LC-CQDs

The cellular uptake mechanism is investigated using pharmacological inhibitors to block some of the major endocytic pathways (Supplementary Fig. 12). The relative cellular uptake percentage is reduced to 62% and 79% when the clathrin and the caveolae mediated endocytosis pathways are inhibited using chlorpromazine and methylated β-cyclodextrin (MβCD), respectively. The relative cellular uptake percentage does not change when macropinocytosis and the polymerization of actin are blocked with CytoD or amiloride. The relative cellular uptake percentage is reduced to 43% when cells are incubated at 4 °C, a temperature at which energy dependent processes can be inhibited. Furthermore, the intracellular trafficking of LC-CQDs is also studied by analyzing the overlapping ratio between LC-CQDs and different subcellular organelles at different incubation duration (Supplementary Figs 13–15). At 30 min, LC-CQDs have a relatively high overlap ratio with early endosome. At 120 min, a large number

of LC-CQDs are in the late endosome and Golgi. At 240 min, most of the LC-CQDs are transported to the Golgi. These results indicate that the uptake of LC-CQDs is an energy-dependent process mediated by clathrin and caveolae; large quantities of LC-CQDs are transported via early endosome, late endosome and eventually to the Golgi through the retrograde trafficking route.

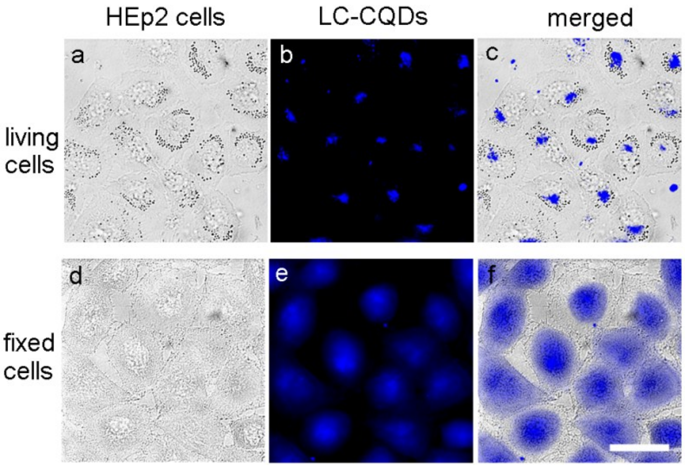

Figure S14. The different staining ability of LC-CQDs for living and fixed cells. (a) Bright field image of living HEp2 cells. (b) The fluorescence image of LC-CQDs in living HEp2 cells. (c) Merged image of a and b. (d) Bright field image of fixed HEp2 cells. (e) The fluorescence image of LC-CQDs in fixed HEp2 cells. (f) Merged image of d and e. Scale bar, 20 μm.

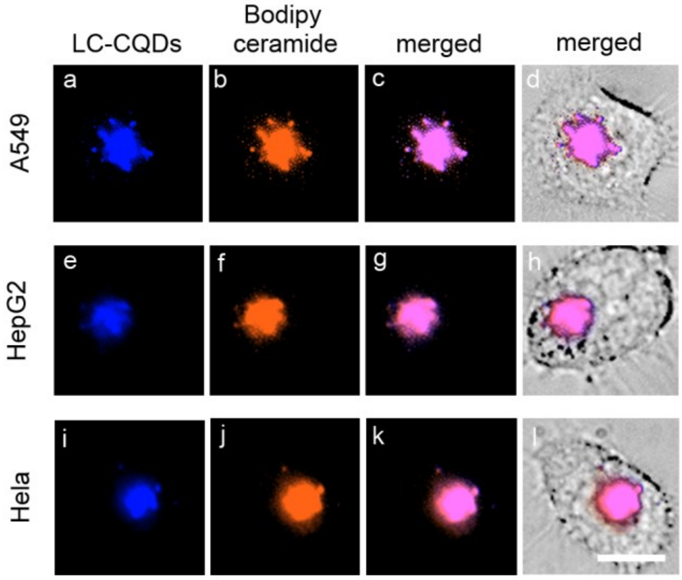

Figure S15. The Golgi targeting ability of LC-CQDs in A549, HepG2, and Hela cell lines. (a) Fluorescence image of LC-CQDs (blue) in A549 cell. (b) Fluorescence image of Bodipy ceramide (red, Golgi dye) in A549 cell. (c) Merged image of a and b. (d) Merged image of a, b, and bright field images. (e) Fluorescence image of LC-CQDs (blue) in HepG2 cell. (f) Fluorescence image of Bodipy ceramide (red, Golgi dye) in HepG2 cell. (g) Merged image of e and f. (h) Merged image of e, f, and bright field images. (i) Fluorescence image of LC-CQDs (blue) in Hela cell. (j) Fluorescence image of Bodipy ceramide (red, Golgi dye) in Hela cell. (k) Merged image of i and j. (l) Merged image of i, j, and bright field images. Scale bar, 10 μm.

Note: In order to investigate whether the Golgi target of LC-CQDs is only for HEp2 cells, other cells lines including A549, HepG2, and Hela cells were incubated with LC-CQDs, respectively. Bodipy ceramide was used to

co-stain these cells after 4 h of incubation of these cells with LC-CQDs. The fluorescent area of LC-CQDs matches very well with those of Bodipy ceramide with a Pearson's correlation factor higher than 0.9 (Figure S14), indicating that preferential accumulation of LC-CQDs in the Golgi has occurred in A549, HepG2, and Hela cell lines.

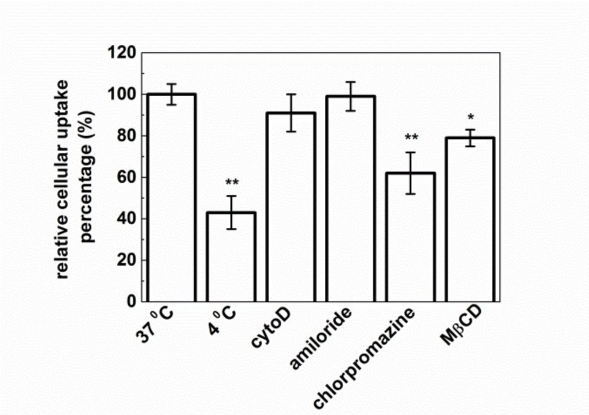

Figure S16. Quantitative analysis of the influence of different uptake inhibitors. **P<0.01; *P<0.05 (one-sided t-test). Error bars represent standard deviations from five replicate experiments. Cyto D: Cytochalasin D; MβCD: Methyl-β-cyclodextran.

Note: These results indicate that the uptake of LC-CQDs is an energy-dependent process mediated by clathrin and caveolae.

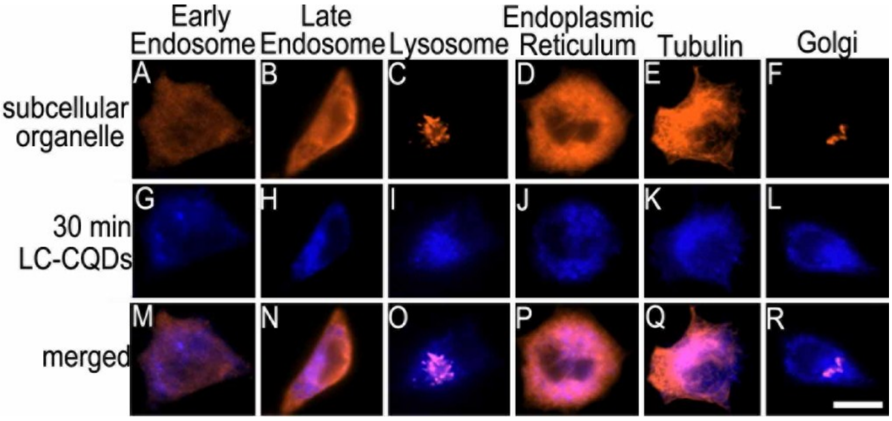

Figure S17. The subcellular distribution of LC-CQDs after the incubation of 30 min. (*A*) Immunofluorescence image of early endosome. The primary antibody was Anti-EEA1. The secondary antibody (red) was goat polyclonal secondary antibody to Rabbit IgG–H&L (Cy3 @) pre-adsorbed. (*B*) Immunofluorescence image of late endosome. The primary antibody was Anti-Rab9. The secondary antibody (red) was goat polyclonal secondary antibody to Rabbit IgG–H&L (Cy3 @) pre-adsorbed. (*C*) Fluorescence image of the lysosome stained by Lyso-Tracker Red. (*D*) Fluorescence image of the endoplasmic reticulum stained by ER-Tracker Red. (*E*) Fluorescence image of the tubulin stained by Tubulin-Tracker Red. (*F*) Fluorescence image of the Golgi stained by Bodipy ceramide. (*G–L*) Fluorescence image of LC-CQDs. (*M–R*) Merged images of fluorescence images. Scale bar, 10 μm.

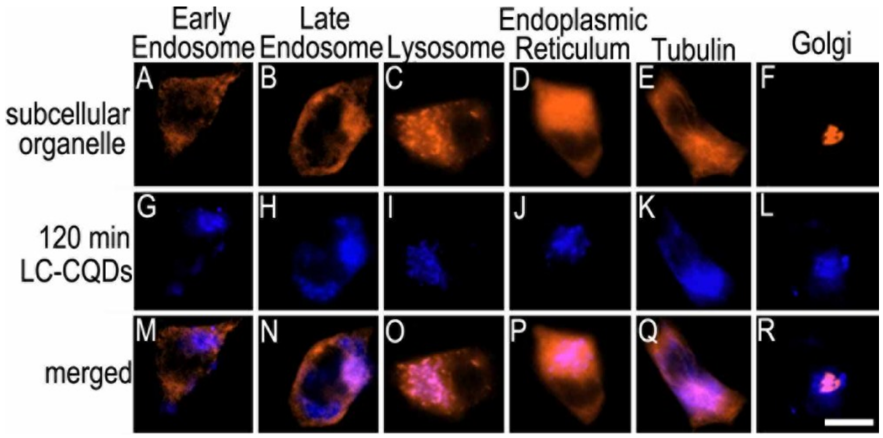

Figure S18. The subcellular distribution of LC-CQDs after the incubation of 120 min. (*A*) Immunofluorescence image of early endosome. The primary antibody was Anti-EEA1. The secondary antibody (red) was goat polyclonal secondary antibody to Rabbit IgG–H&L (Cy3 [@]) pre-adsorbed. (*B*) Immunofluorescence image of late endosome. The primary antibody was Anti-Rab9. The secondary antibody (red) was goat polyclonal secondary antibody to Rabbit IgG–H&L (Cy3 [@]) pre-adsorbed. (*C*) Fluorescence image of the lysosome stained by Lyso-Tracker Red. (*D*) Fluorescence image of the endoplasmic reticulum stained by ER-Tracker Red. (*E*) Fluorescence image of the tubulin stained by Tubulin-Tracker Red. (*F*) Fluorescence image of the Golgi stained by Bodipy ceramide. (*G*–*L*) Fluorescence image of LC-CQDs. (*M*–*R*) Merged images of fluorescence images. Scale bar, 10 μm.

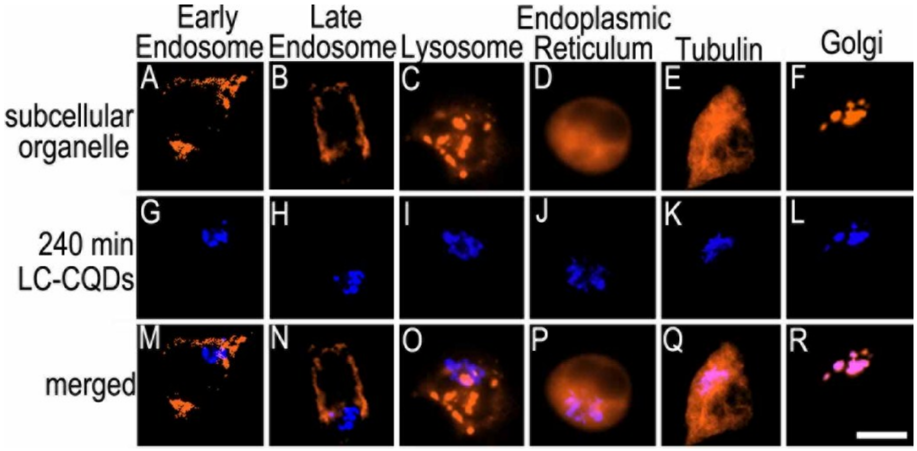

Figure S19. The subcellular distribution of LC-CQDs after the incubation of 120 min. (*A*) Immunofluorescence image of early endosome. The primary antibody was Anti-EEA1. The secondary antibody (red) was goat polyclonal secondary antibody to Rabbit IgG–H&L (Cy3 [@]) pre-adsorbed. (*B*) Immunofluorescence image of late endosome. The primary antibody was Anti-Rab9. The secondary antibody (red) was goat polyclonal secondary antibody to Rabbit IgG–H&L (Cy3 [@]) pre-adsorbed. (*C*) Fluorescence image of the lysosome stained by Lyso-Tracker Red. (*D*) Fluorescence image of the endoplasmic reticulum stained by ER-Tracker Red. (*E*) Fluorescence image of the tubulin stained by Tubulin-Tracker Red. (*F*) Fluorescence image of the Golgi stained by Bodipy ceramide. (*G*–*L*) Fluorescence image of LC-CQDs. (*M*–*R*) Merged images of fluorescence images. Scale bar, 10 μm.

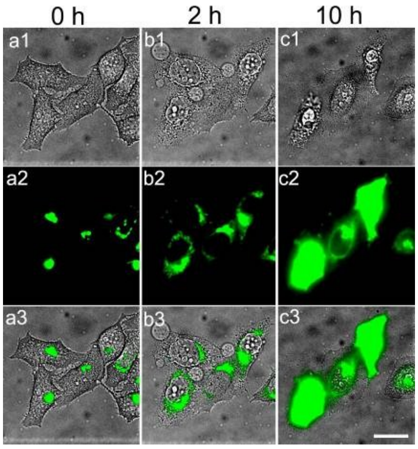

Figure S20. Rupture and de-attachment change of cell morphology of HEp2 cells occurred after the stain of NBD C6-ceramide (1 μM, which is largely lower than the typically used concentration of 5 μM). In the incubation medium, 1 μM NBD C6-ceramide was kept still. Scale bar, 20 μm. Images are representative of replicate experiments (*n* = 5).

Note: The results suggest that NBD C6-ceramide, which has been widely used for the Golgi stain, is not suitable for the long-term imaging of the Golgi.

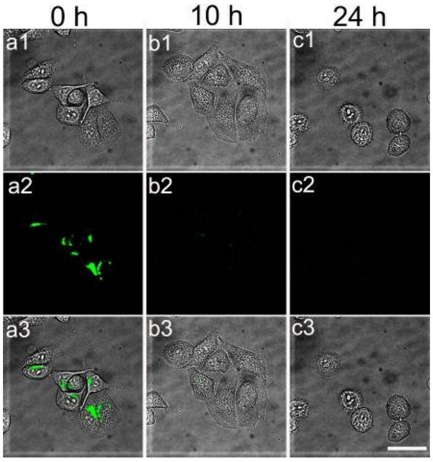

Figure S21. Rupture and de-attachment change of cell morphology of HEp2 cells after the stain of NBD C_6-ceramide (5 μM, which is the typically used concentration). In the incubation medium, no further addition of NBD C_6-ceramide was made. Scale bar, 20 μm. Images are representative of replicate experiments (*n* = 5).

Notes: the morphological hallmarks of cell death, bleb formation, cell body shrinkage, and fragmentation were observed in HEp2 cells after the staining of the Golgi by NBD C_6-ceramide. On the other hand, the fluorescence images show that NBD C_6-ceramide disappeared or widely distributed in cells as time went by. These observations suggest a needs for caution in using ceramide analogues in long-term experiments.

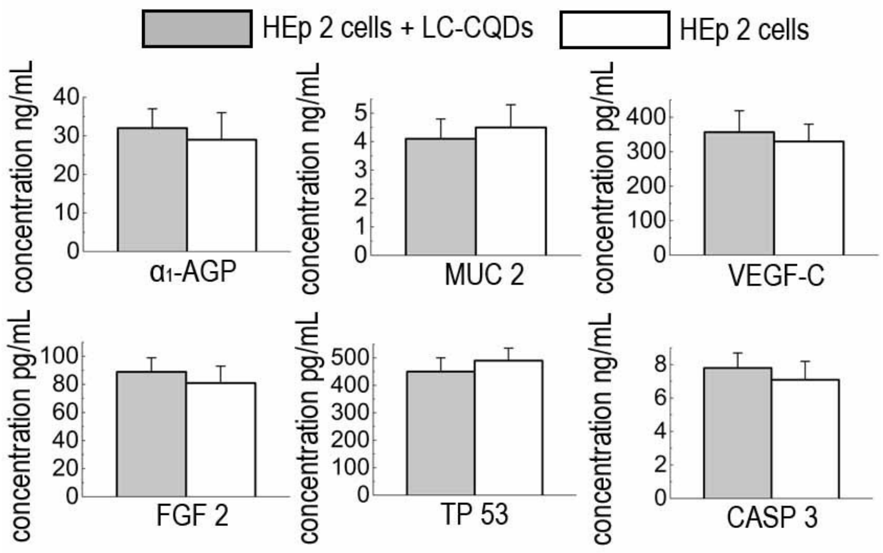

Figure S22. protein concentrations of HEp2 cells incubated with or without LC-CQDs. concentrations are quantified using commercially available ELISAs. Error bars represent standard deviations from three replicate experiments. α_1-AGP: Alpha-1-Acid Glycoprotein; MUC 2: Mucin 2; VEGF-C: Vascular Endothelial Growth Factor C); FGF 2: Basic Fibroblast Growth Factor; TP53: Tumor Protein p53; CASP 3: Caspase 3.

Note: The results suggest that the quantity of secreted proteins, glycoproteins and apoptosis proteins were not affected when cells are treatment with LC-CQDs (1 mg mL^{-1}), indicating the good biocompatibility of LC-CQDs and the possibility for *in situ* long-term imaging.

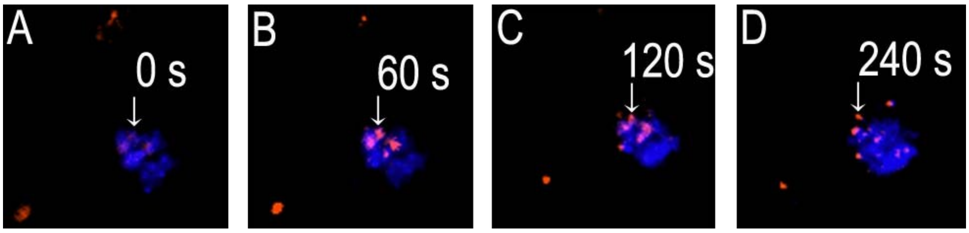

Figure S23. The transportation of quantum dots (red) in the Golgi. The images are representative of replicate experiments (n = 5).

Note: The results suggest that cellular transportation is not affected when the Golgi is stained with LC-CQDs, indicating the good biocompatibility of LC-CQDs and the possibility for *in situ* long-term imaging.

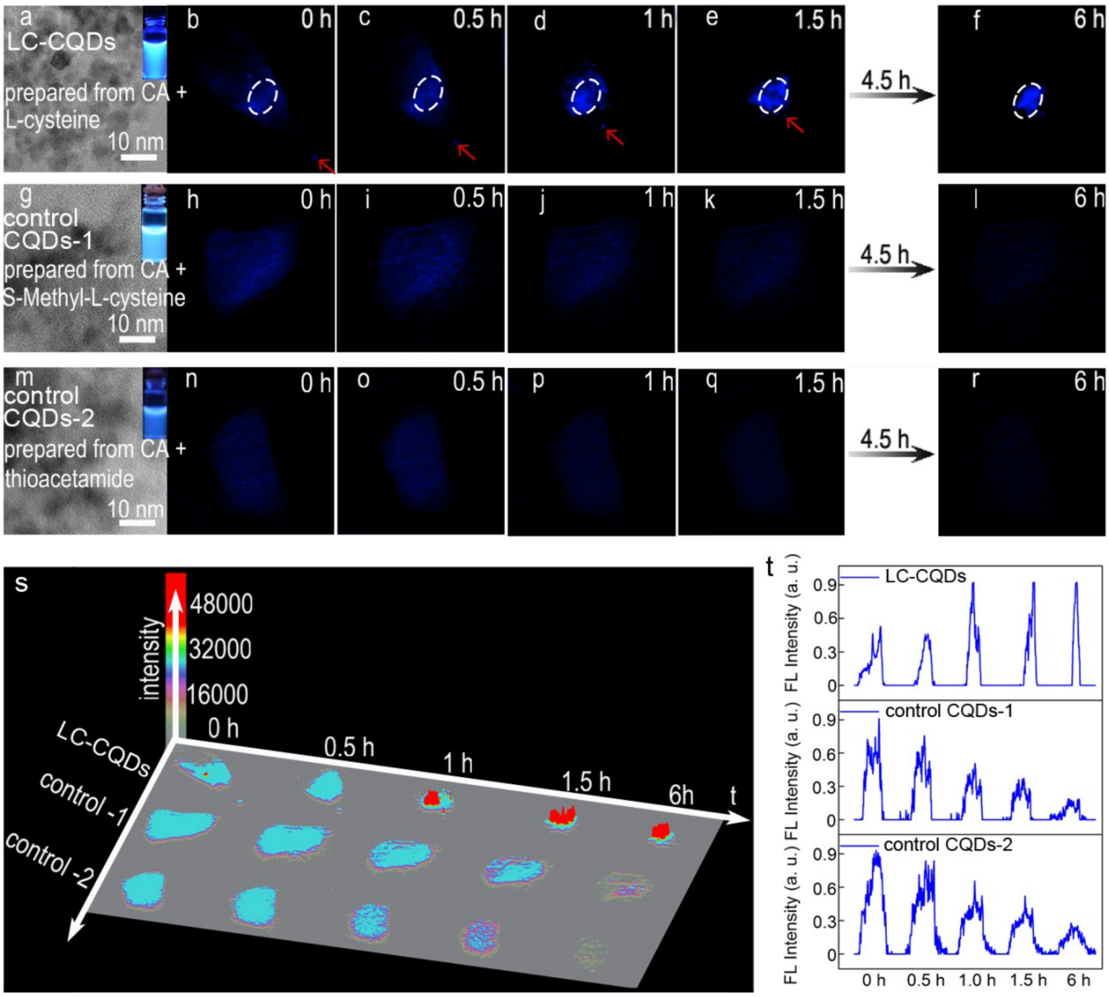

Figure S24. Changes in the cellular distribution of different kinds of carbon quantum dots (CQDs). (a) TEM image of LC-CQDs. Inset: Photographs of LC-CQDs under illumination of UV (365 nm) light. (b-f) Fluorescence images of a certain amount of LC-CQDs in the same cell at different time; Red arrows point to the location of a small amount of LC-CQDs in the cell at different time. (g) TEM image of control CQDs -1 (prepared from citric acid and S-Methyl-L-cysteine). Inset: Photographs of control CQDs -1 under illumination of UV (365 nm) light. (h-l) Fluorescence images of a certain amount of CQDs -1 in the same cell at different time; (m) TEM image of control CQDs -2 (prepared from citric acid and thioacetamide). Inset: Photographs of control CQDs -2 under illumination of UV (365 nm) light. (n-r) Fluorescence images of a certain amount of CQDs -2 in the same cell at different time; (s) surface plot of intracellular fluorescence intensity of different kinds of carbon dots at different time; (t) changes of fluorescence intensity of different kinds of carbon dots at different time. The images are representative of replicate experiments (n = 5).

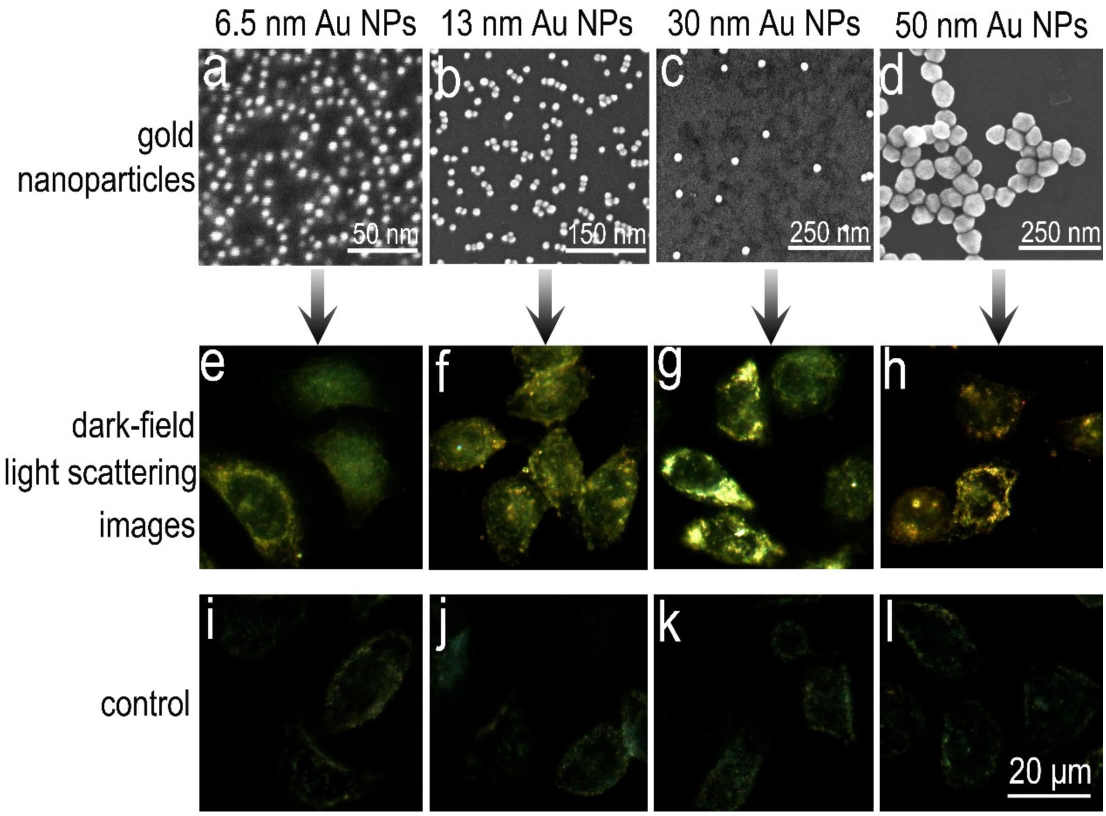

Figure S25. The dark-field light scattering images of gold nanoparticles with different sizes in HEp2 cells. (**a-d**) Scanning electron microscope (SEM) images of gold nanoparticles with different sizes; (**e-h**) the dark-field light scattering images of gold nanoparticles with different sizes; (**i-l**) the dark-field light scattering images of HEp2 cells incubated without gold nanoparticles. The images are representative of replicate experiments (n = 5).

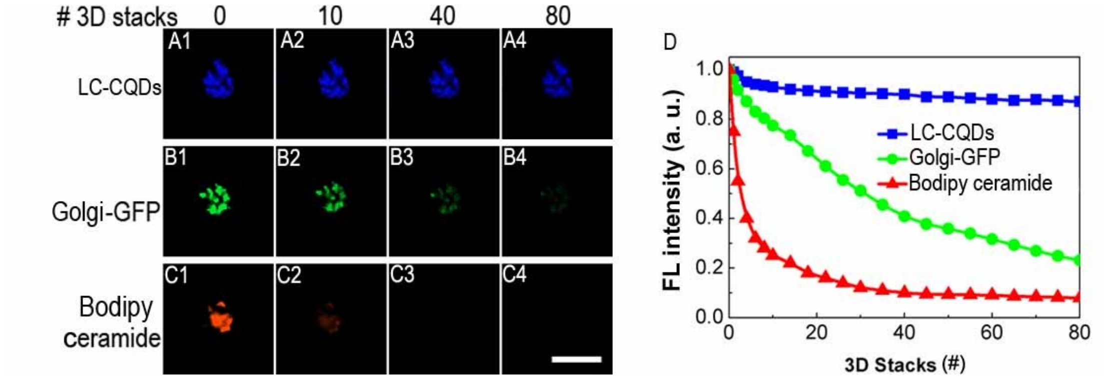

Figure S26. Golgi labeled with LC-CQDs are extremely stable to prolonged illumination. (*A*) Images show cells labeled with LC-CQDs, Golgi-GFP, or Bodipy ceramide after acquisition of 1-80 3D image stacks (30 images per stack). (*B*) Plot of the relative, average per-cell intensity of cells labeled with different Golgi probes as a function of the number of acquired 3D stacks. Scale bar, 5 μm. The images are representative of replicate experiments (*n* = 5).

Note: For Bodipy ceramide and Golgi-GFP labeling, 70% and 90% loss of fluorescence after 80 3D 'stacks' are observed, respectively. As a clear contrast, the fluorescent signal of cells treated with LC-CQDs decreases by only 10% after same number of 3D 'stacks'. These results demonstrate that LC-CQDs present very good photostability than Golgi-GFP and Bodipy ceramide in living cells, thus are feasible for the *in situ* long-term fluorescence observation of the Golgi.

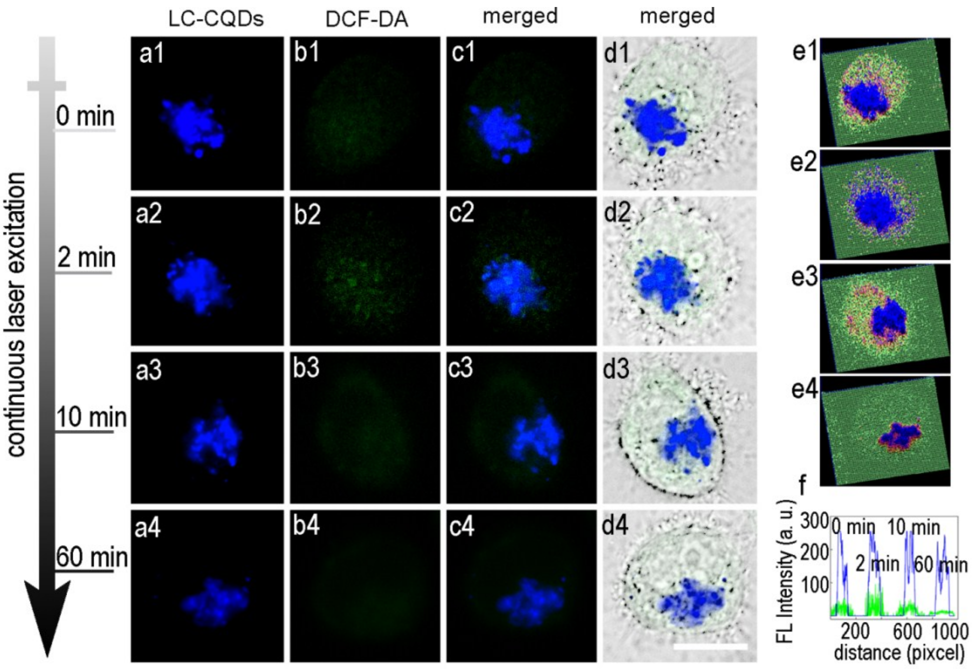

Figure S27. The DCF-DA assay within cells in the process of continuous laser excitation. (a) Photostability of LC-CQDs in cells under continuous laser excitation. (b) The fluorescence of DCF-DA within cells after the continuous excitation of LC-CQDs at different time. (c) Merged images of a and b. (d) merged images of a, b, and bright field images. Scale bar, 10 μm. (e) The corresponding three-dimensional mode of the fluorescence images showed in c1, c2, c3, and c4, respectively. (f) Quantitative analysis of the fluorescence intensity of LC-CQDs and DCF-DA in the process of laser excitation.

Note: In order to investigate the oxygen species generation ability of LC-CQDs within cells under continuous laser excitation, we used 2',7'-dichlorofluorescin diacetate (DCF-DA) for sensing the generation of reactive oxygen species. As shown in Figure S27, only weak green fluorescence was observed after the continuous excitation of LC-CQDs. The weak green fluorescein would not turn bright green with the increasing of exciting time. The results indicate that LC-CQDs may generate very few reactive oxygen species in the process of the continuous excitation. The weak ability of reactive oxygen species generation of LC-CQDs may attribute to the neutral surface charge (-6 mV) and cysteine residues on the surface of LC-CQDs. Havrdova *et al.* reported that carbon dots could not generate reactive oxygen species if their surface charge is neutral (doi:10.1016/j.carbon.2015.12.027). Furthermore, many scientists have found that cysteine could reduce oxidative stress (doi:10.1038/35041687). The free thiol groups on the surface of LC-CQDs may act as peroxide scavengers within cells. We may draw the conclusion that LC-CQDs would not generate reactive oxygen species in long-term in situ Golgi imaging.

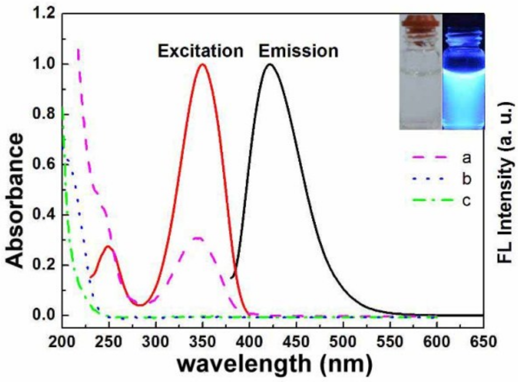

Figure S28. Fluorescence spectra (solid curves) and UV/Vis absorption spectra (dot curves) of DC-CQDs (the enantiomer of LC-CQDs, prepared from D-cysteine and citric acid). a, DC-CQDs; b, citric acid; c, D-cysteine. Inset: carbon quantum dots under illumination of white light (left) and UV (365 nm) light (right). The results indicate the formation of DC-CQDs.

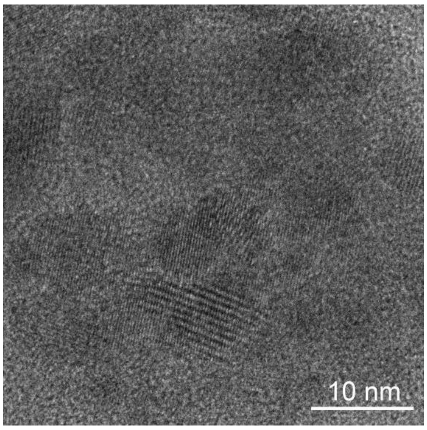

Figure S29. TEM image of the carbon quantum dots prepared from citric acid and D-cysteine. The results indicate the formation of DC-CQDs.

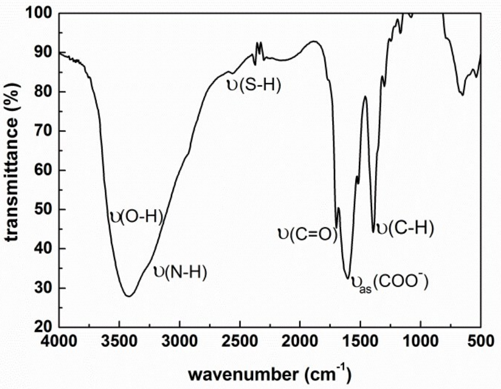

Figure S30. FTIR spectrum of the carbon quantum dots prepared from citric acid and D-cysteine. The results indicate the formation of DC-CQDs.

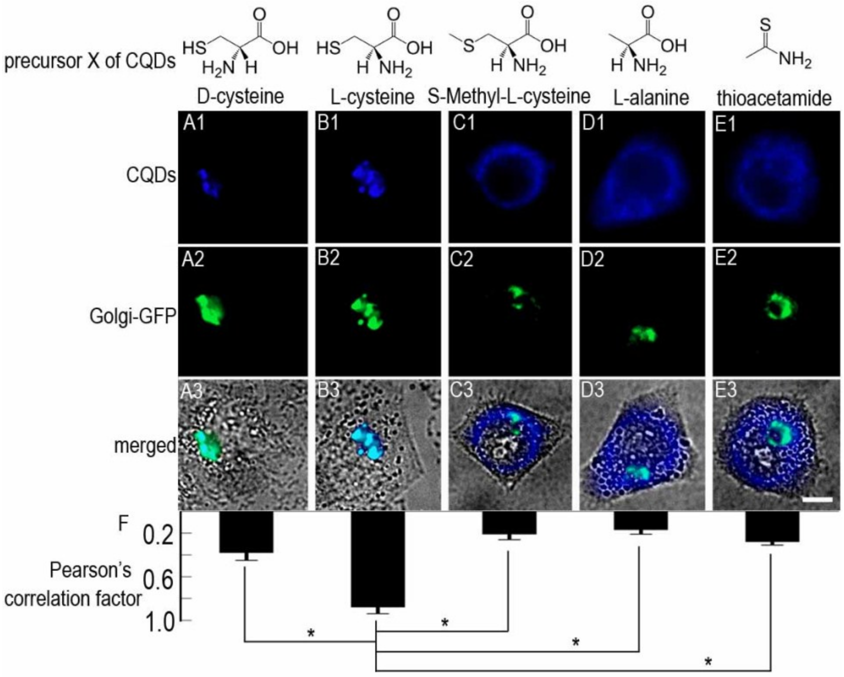

Figure S31. The Golgi imaging ability of other carbon quantum dots. Images of cellular uptake for five different kinds of CQDs prepared from citric acid and X (X = D-cysteine, L-cysteine, S-methyl-L-cysteine, L-alanine, thioacetamide). (*A*1–*E*1) Fluorescence images of different CQDs (blue). (*A*2–*E*2) Fluorescence images of Golgi-GFP (green, Golgi specific GFP). (*A*3–*E*3) Merged fluorescence and bright field images. Scale bar, 10 µm. (*F*) Pearson's correlation factor between Golgi-GFP and CQDs prepared using different carbon sources in images *A*–*E*, respectively, calculated by Image-Pro Plus 6.0. Error bars represent standard deviations from five replicate experiments.*$P<0.05$ (one-sided t-test).

Note: The results indicate that L-type stereo configuration and free thiol groups are indispensable for the Golgi targeting of LC-CQDs.

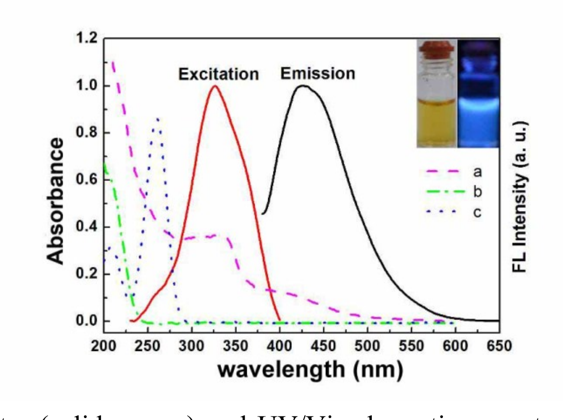

Figure S32. Fluorescence spectra (solid curves) and UV/Vis absorption spectra (dot curves) of carbon quantum dots prepared from thioacetamide and citric acid. a, carbon quantum dots; b, citric acid; c, thioacetamide. Inset: Photograph of carbon quantum dots under illumination of white light (left) and UV (365 nm) light (right). The results indicate the formation of thioacetamide carbon quantum dots which have no L-type stereo configuration.

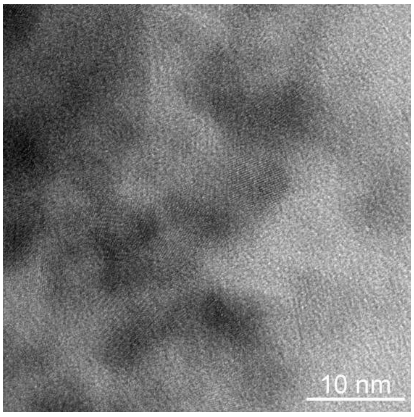

Figure S33. TEM image of the carbon quantum dots prepared from citric acid and thioacetamide. The results indicate the formation of thioacetamide carbon quantum dots which have no L-type stereo configuration.

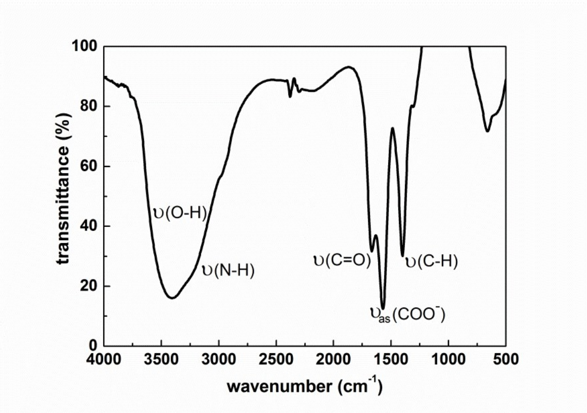

Figure S34. FTIR spectrum of the carbon quantum dots prepared from citric acid and thioacetamide. The results indicate the formation of thioacetamide carbon quantum dots which have no L-type stereo configuration.

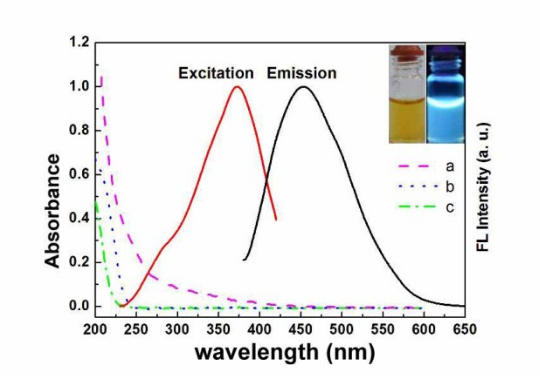

Figure S35. Fluorescence spectra (solid curves) and UV/Vis absorption spectra (dot curves) of carbon quantum dots prepared from S-Methyl-L-cysteine and citric acid. a, carbon quantum dots; b, citric acid; c, S-Methyl-L-cysteine. Inset: Photograph of carbon quantum dots under illumination of white light (left) and UV (365 nm) light (right). The results indicate the formation of S-Methyl-L-cysteine carbon quantum dots which have no free thiol groups.

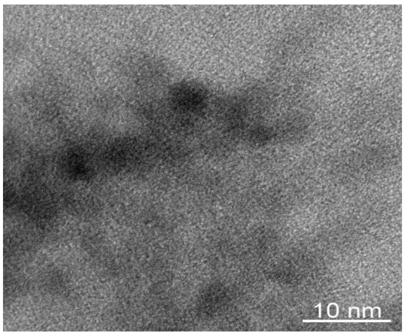

Figure S36. TEM image of the carbon quantum dots prepared from citric acid and S-Methyl-L-cysteine. The results indicate the formation of S-Methyl-L-cysteine carbon quantum dots which have no free thiol groups.

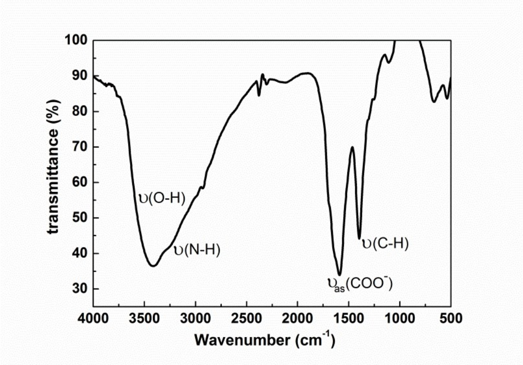

Figure S37. FTIR spectrum of the carbon quantum dots prepared from citric acid and S-Methyl-L-cysteine. The results indicate the formation of S-methyl-L-cysteine carbon quantum dots which have no free thiol groups.

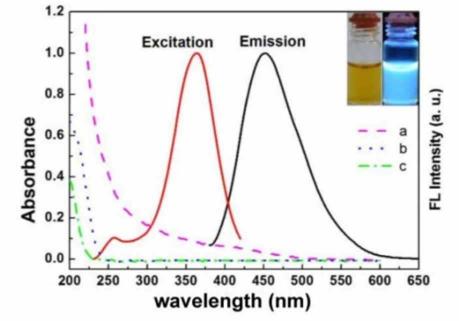

Figure S38. Fluorescence spectra (solid curves) and UV/Vis absorption spectra (dot curves) of carbon quantum dots prepared from L-analnine and citric acid. a, carbon quantum dots; b, citric acid; c, L-analnine. Inset: Photograph of carbon quantum dots under illumination of white light (left) and UV (365 nm) light (right). The results indicate the formation of L-analnine carbon quantum dots which have no free thiol groups.

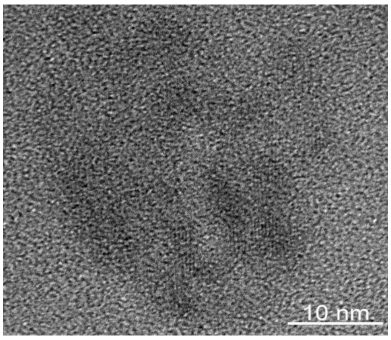

Figure S39. TEM image of the carbon quantum dots prepared from citric acid and L-analnine. The results indicate the formation of L-analnine carbon quantum dots which have no free thiol groups.

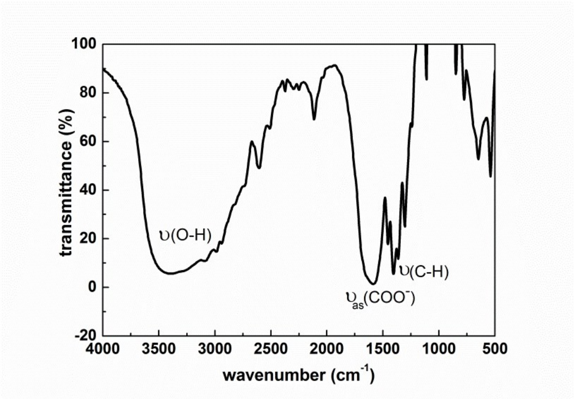

Figure S40. FTIR spectrum of the carbon quantum dots prepared from citric acid and L-analnine. The results indicate the formation of L-analnine carbon quantum dots which have no free thiol groups.

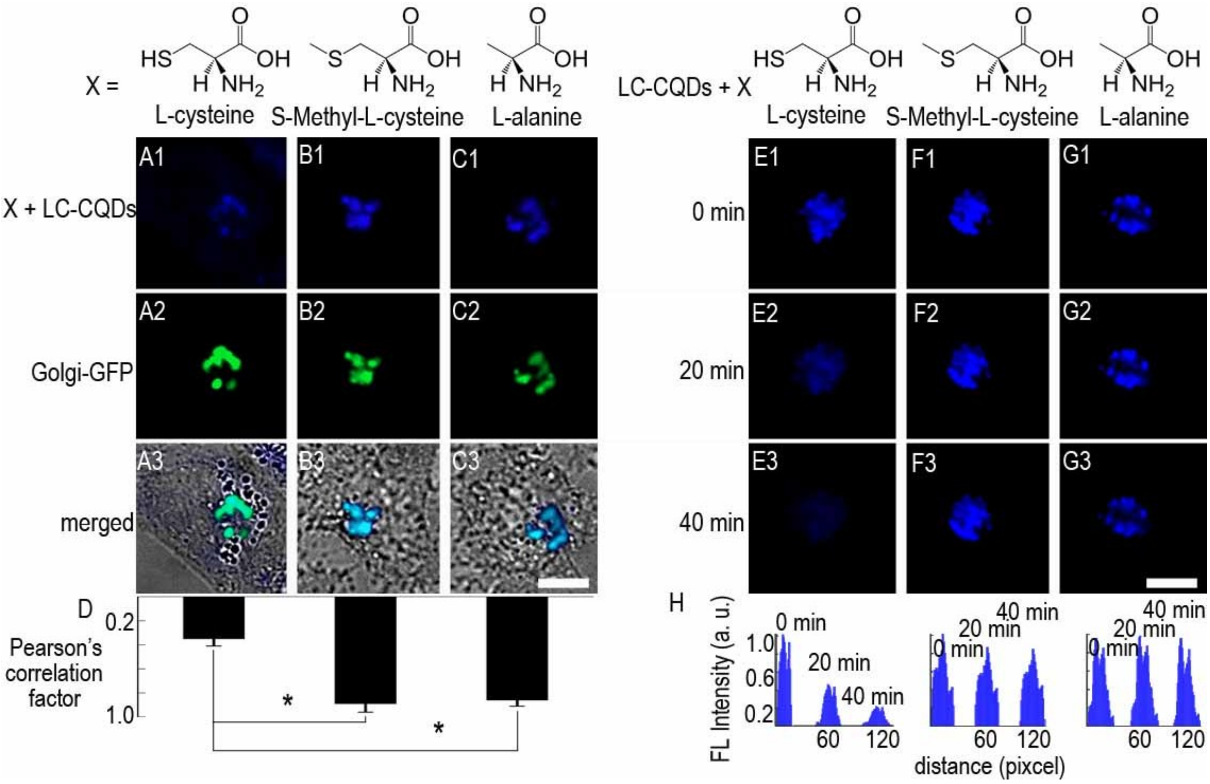

Figure S41. The competitive inhibition of targeting ability of LC-CQDs. (*A–C*) The cells were incubated with X (0.5 mM) before the addition of LC-CQDs. (*A*1–*C*1) Fluorescence images of LC-CQDs (blue). (*A*2–*C*2) Fluorescence images of Golgi-GFP (Green, Golgi specific GFP). (*A*3–*C*3) Merged fluorescence and bright field images. (*D*) Pearson's correlation factors between Golgi-GFP and LC-CQDs in images *A*, *B* and *C*, respectively, calculated by Image-Pro Plus 6.0. Error bars represent standard deviations from five replicate experiments.*P<0.05 (one-sided t-test). (*E–G*) The fluorescence change process in Golgi after the addition of X (0.1 mM). Scale bar, 10 μm. (*H*) The change of fluorescence intensity of LC-CQDs incubated with X, calculated by Image-Pro Plus 6.0.

Note: Competitive inhibition experiments were conducted to identify the binding site of LC-CQDs. It is found that the targeting ability of LC-CQDs is inhibited with the Pearson's correlation factor of 0.35, if cells were incubated with cysteine before the addition of LC-CQDs (Supplementary Fig. 34 *A* and *D*). Neither S-methyl-L-cysteine nor L-alanine shows any of such inhibitory effects (Supplementary Fig. 34 *B–D*) with the Pearson's correlation factors of 0.89 and 0.86, respectively. These results suggest that the free thiol group of L-cysteine is capable of inhibiting the Golgi targeting ability of LC-CQDs. Fluorescence in the Golgi disappears if cells are stained first with LC-

CQDs and then incubated with L-cysteine (Supplementary Fig. 34 *E* and *H*). Such competitive binding effects, however, are not observed for S-methyl-L-cysteine or L-alanine (Supplementary Fig. 34 *F–H*), demonstrating the importance of free thiol groups in the detachment of LC-CQDs from the Golgi. The competitive binding between L-cysteine and LC-CQDs toward the Golgi suggests that LC-CQDs may bind to the sulfhydryl receptor site of the Golgi through the formation of disulphide bonds in the oxidizing environment of the Golgi lumen.

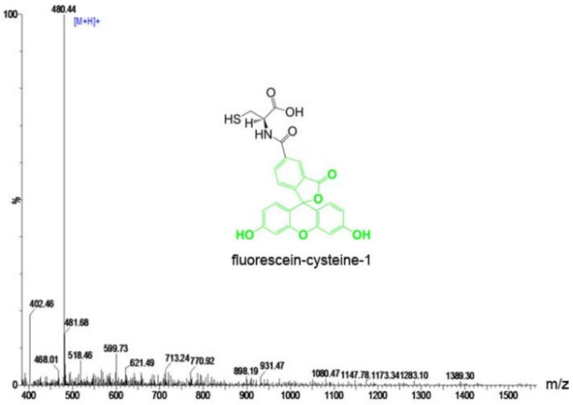

Figure S42. Mass spectroscope of fluorescein-cysteine-1. Probe: ESI. Cone: 50 V. Capillary: 3.00 KV. Extractor: 5 V. Gas Flow: 350 L/h. T. Flow:0.2ml/min. Desolvation Temp: 350 ^{o}C. B. conc: 50% H_2O/50% ACN. The results indicate the formation of fluorescence-cysteine-1.

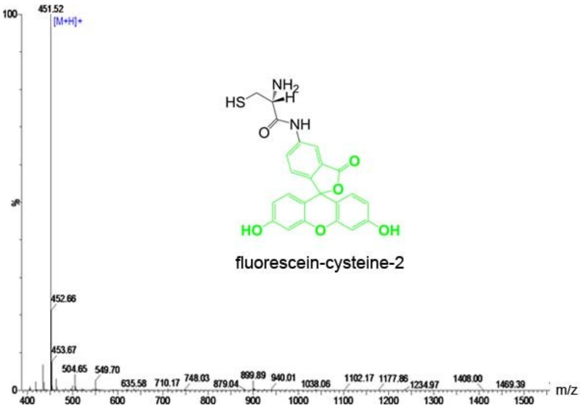

Figure S43. Mass spectroscope of fluorescein-cysteine-2. Probe: ESI. Cone: 50 V. Capillary: 3.00 KV. Extractor: 5 V. Gas Flow: 350 L/h. T. Flow:0.2ml/min. Desolvation Temp: 350 ^{o}C. B. conc: 50% H_2O/50% ACN. The results indicate the formation of fluorescence-cysteine-2.

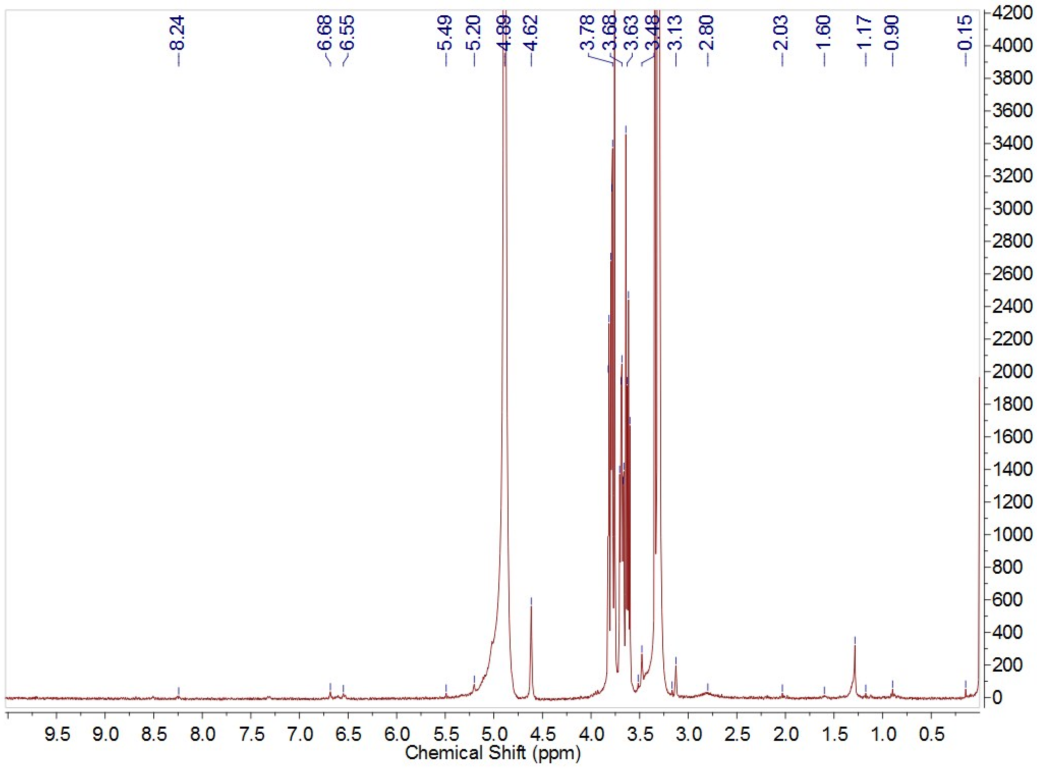

Figure S44. ^{1}H NMR spectrum of fluorescein-cysteine-1 in CD_3OD. δ (ppm) = 8.24 (Ar-O$\underline{H}$), 6.68 and 6.55 (Ar-$\underline{H}$), 4.62 (-CO-N$\underline{H}$-) 3.75 (-NH-C$\underline{H}$(CH_2SH)-COOH), 3.63 (-CH-C$\underline{H}_2$-SH), 1.17 (-S$\underline{H}$). The strong peak in 4.89 should be attributed to H_2O.

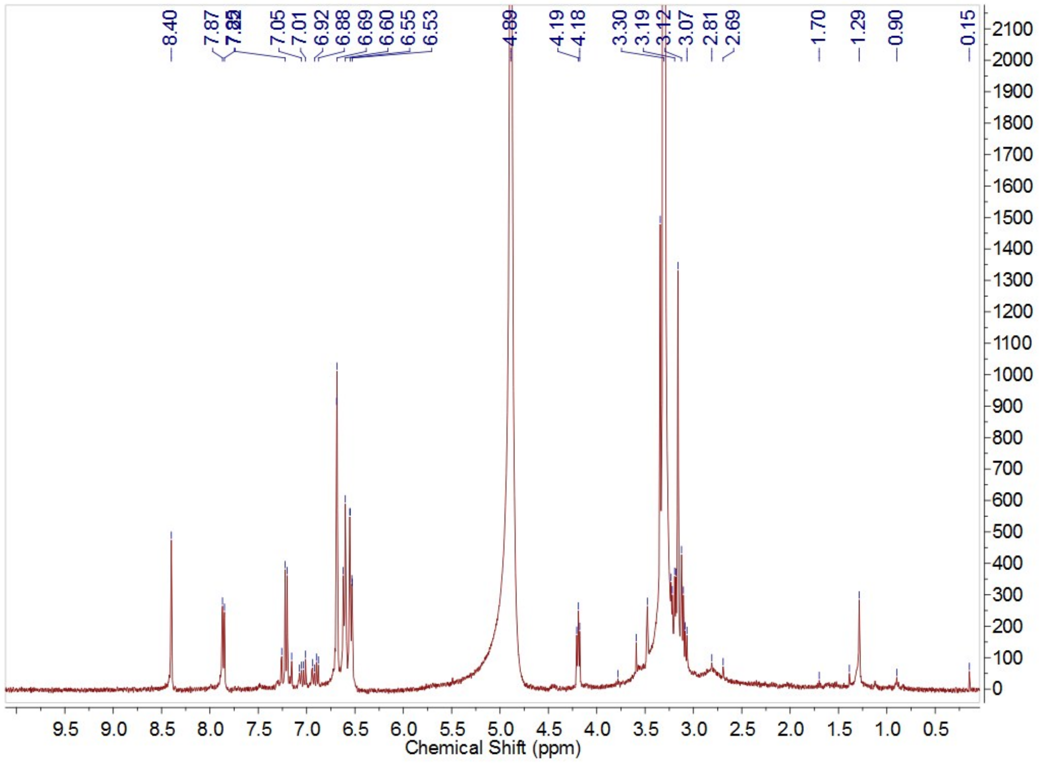

Figure S45. ^{1}H NMR spectrum of fluorescein-cysteine-2 in CD_3OD. δ (ppm) = 8.40 (Ar-O*H*), 7.87 (Ar-N*H*-CO-), 7.50-6.50 (Ar-*H*), 3.73 (NH_2-C*H*(CH_2SH)-CO-), 3.48 (-CH-CH_2-SH), 3.12 (-NH_2), 1.29 (-S*H*). The strong peak in 4.89 should be attributed to H_2O.

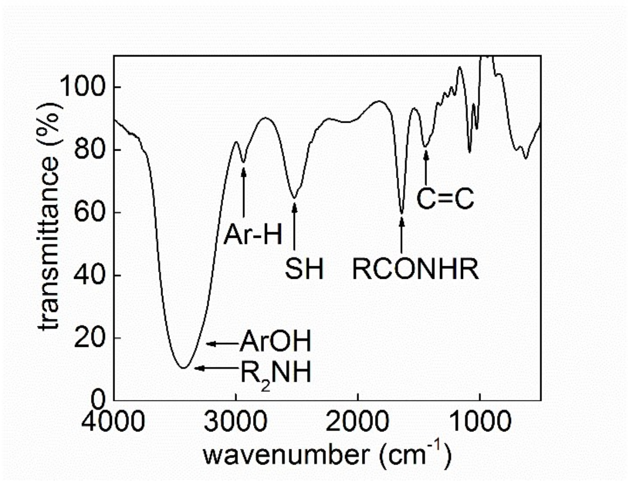

Figure S46. FTIR spectrum of fluorescein-cysteine-1.

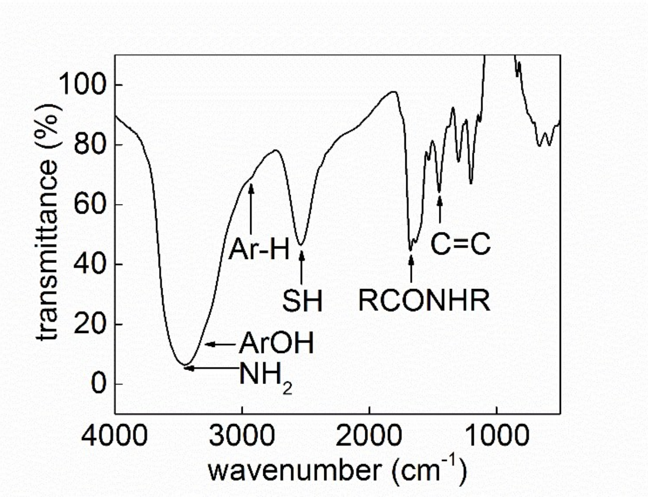

Figure S47. FTIR spectrum of fluorescein-cysteine-2.

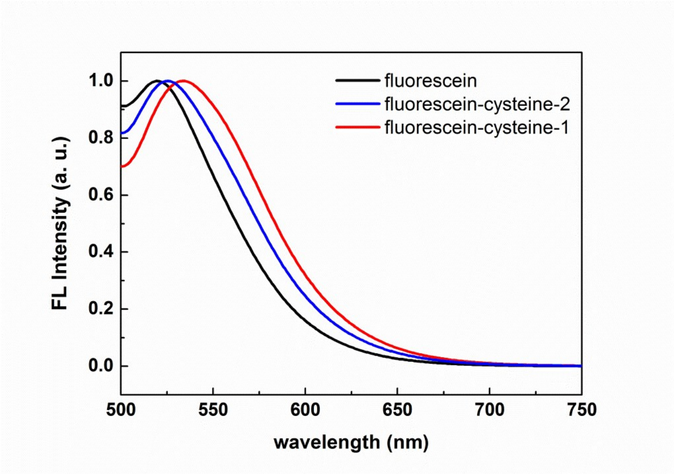

Figure S48. Fluorescence emission spectra of fluorescein (black line), fluorescein-cysteine-1 (blue line), and fluorescein-cysteine-2 (red line). The results indicate L-cysteine has been linked with fluorescein.

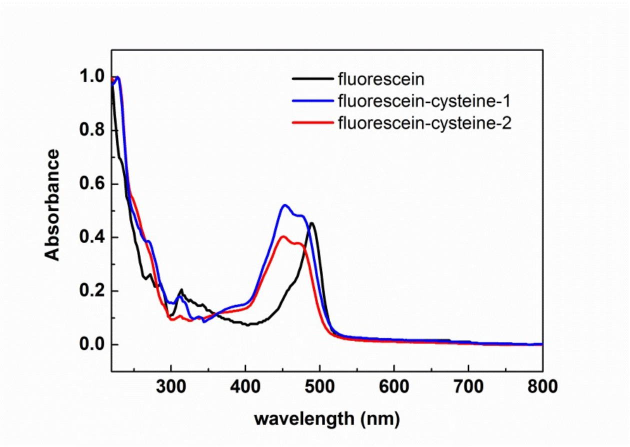

Figure S49. UV/Vis absorption spectra of fluorescein (black line), fluorescein-cysteine-1 (blue line), and fluorescein-cysteine-2 (red line). The results indicate L-cysteine has been linked with fluorescein.

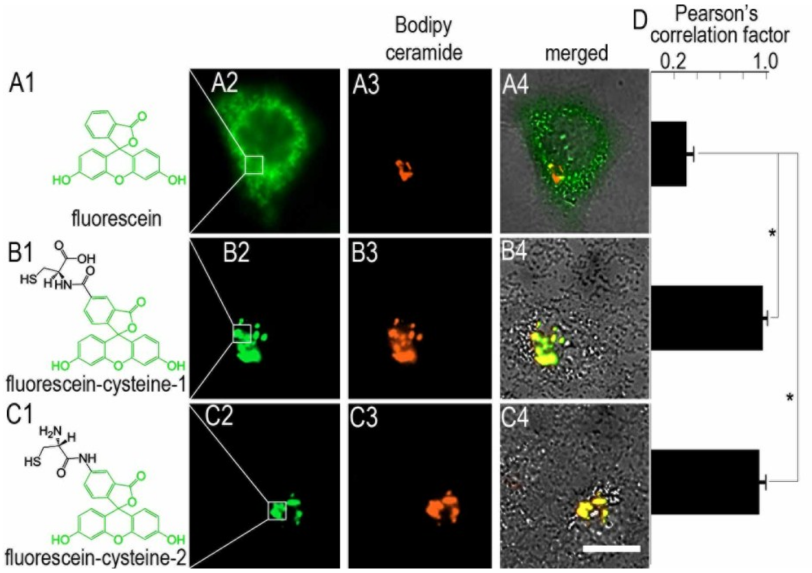

Figure S50. The Golgi targeting ability of cysteine. (*A*1–*C*1) Chemical structures of fluorescein, fluorescein-cysteine-1, and fluorescein-cysteine-2. (*A*2–*C*2) Fluorescence images of fluorescein, fluorescein-cysteine-1, where the binding group of cysteine was amino, and fluorescein-cysteine-2, where the binding group of cysteine was carboxyl. (*A*3–*C*3) Fluorescence images of Bodipy ceramide (red, Golgi specific dye). (*A*4–*C*4) Merged fluorescence and bright field images. Scale bar, 10 μm. (*E*) Pearson's correlation factor between fluorescence-cysteine dyes and Bodipy ceramide. , calculated by Image-Pro Plus 6.0. Error bars represent standard deviations from five replicate experiments.**P*<0.05 (one-sided *t*-test).

Note: The results indicate that L-cysteine is an effective agent for the Golgi targeting.

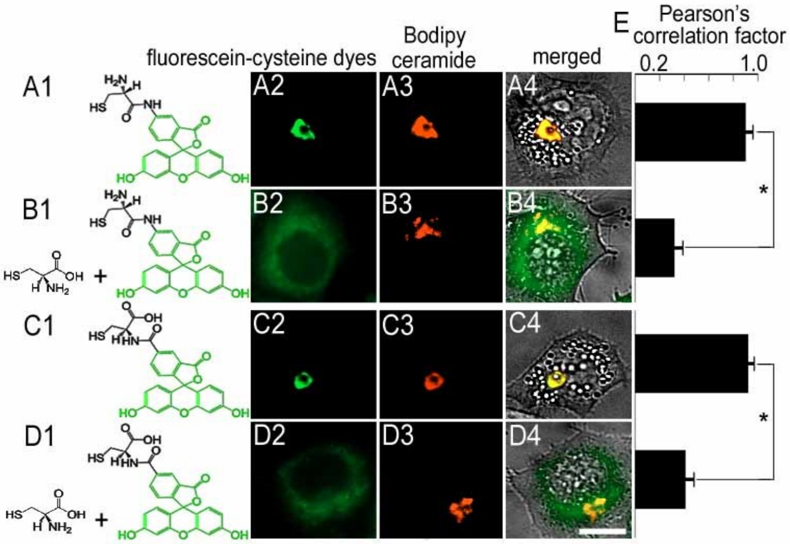

Figure S51. The competitive inhibition for targeting ability of fluorescein-cysteine dyes. (*A*) The cells were not incubated with cysteine (0.5 mM) before the addition of fluorescein-cysteine-2. (*B*) The cells were incubated with cysteine (0.5 mM) before the addition of fluorescein-cysteine-2. (*C*) The cells were not incubated with cysteine (0.5 mM) before the addition of fluorescein-cysteine-1. (*D*) The cells were incubated with cysteine (0.5 mM) before the addition of fluorescein-cysteine-1. Scale bar, 10 μm. (*E*) Pearson's correlation factor between fluorescence-cysteine dyes and Bodipy ceramide, calculated by Image-Pro Plus 6.0. Error bars represent standard deviations from five replicate experiments.**P*<0.05 (one-sided *t*-test).

Note: The results indicate that L-cysteine is an effective inhibitor for the Golgi targeting of fluorescein-cysteine complexes, which may bind on the cysteine binding site of the Golgi lumen.

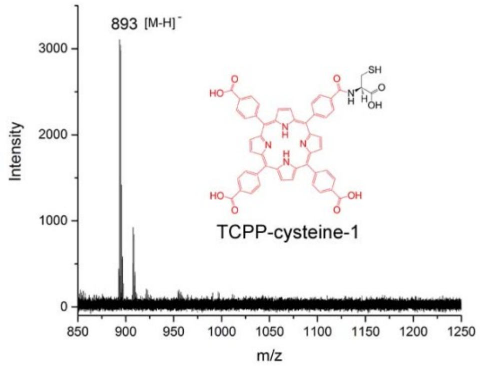

Figure S52. Matrix-assisted laser desorption/ ionization time of flight mass spectrometry (MALDI-TOF MS) analysis of TCPP-cysteine-1. The results indicate the formation of TCPP-cysteine-1.

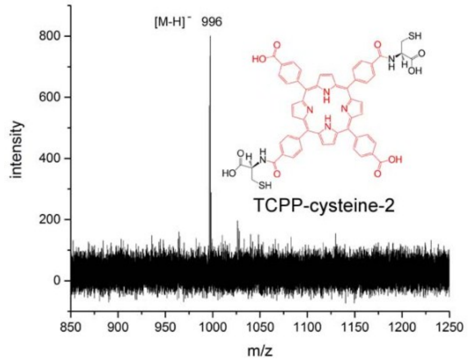

Figure S53. Matrix-assisted laser desorption/ ionization time of flight mass spectrometry (MALDI-TOF MS) analysis of TCPP-cysteine-2. The results indicate the formation of TCPP-cysteine-1.

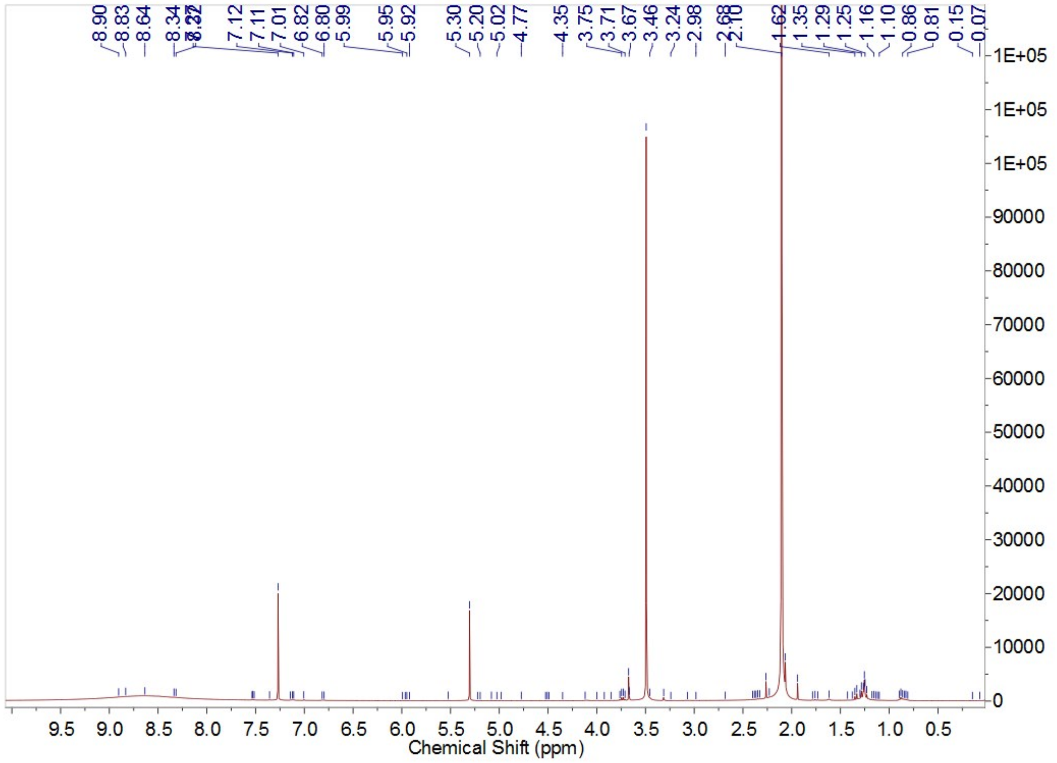

Figure S54. ^{1}H NMR spectrum of TCPP-cysteine-1 in $CDCl_3$. δ (ppm) = 8.64 (β H in the pyrrole ring of TCPP), 7.70-6.70 (Ar-*H*), 5.20 (-CO-N*H*-), 3.71 (-NH-C*H*(CH_2SH)-CO-), 3.46 (-CH-CH_2-SH), 1.25 (-S*H*).

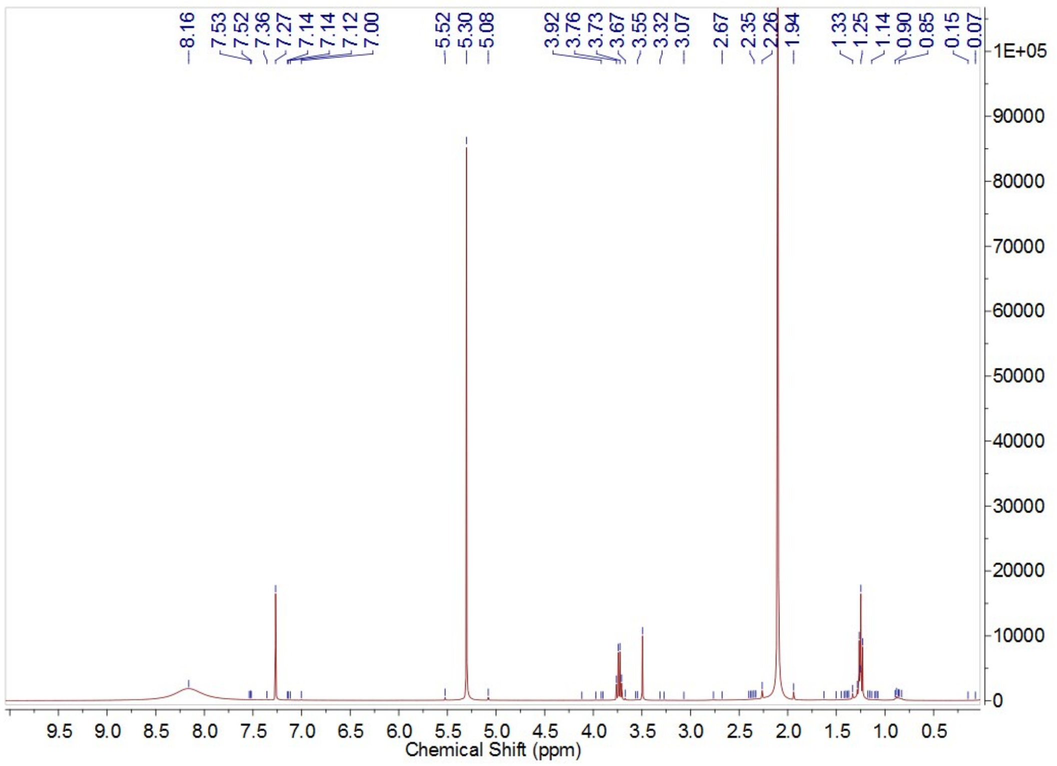

Figure S55. ^{1}H NMR spectrum of TCPP-cysteine-2 in $CDCl_3$. δ (ppm) = 8.16 (β H in the pyrrole ring of TCPP), 7.60-7.00 (Ar-*H*), 5.30 (-CO-N*H*-), 3.73 (-NH-C*H*(CH_2SH)-CO-), 3.55 (-CH-CH_2-SH), 1.25 (-S*H*).

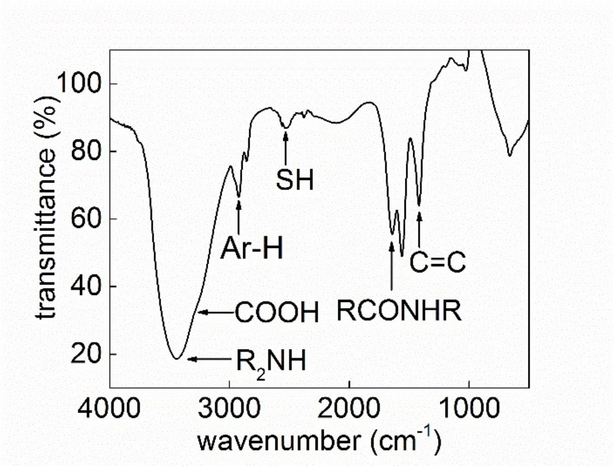

Figure S56. FTIR spectrum of TCPP-cysteine-1.

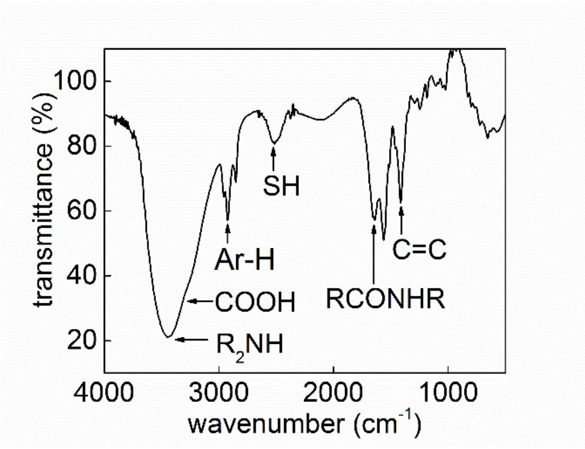

Figure S57. FTIR spectrum of TCPP-cysteine-2.

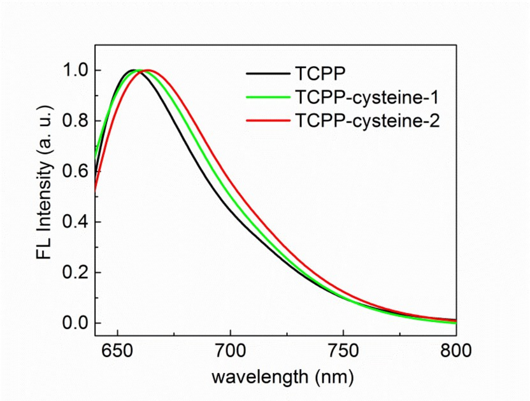

Figure S58. Fluorescence emission spectra of TCPP (black line), TCPP-cysteine-1 (green line), and TCPP-cysteine-2 (red line). The results indicate L-cysteine has been linked with TCPP.

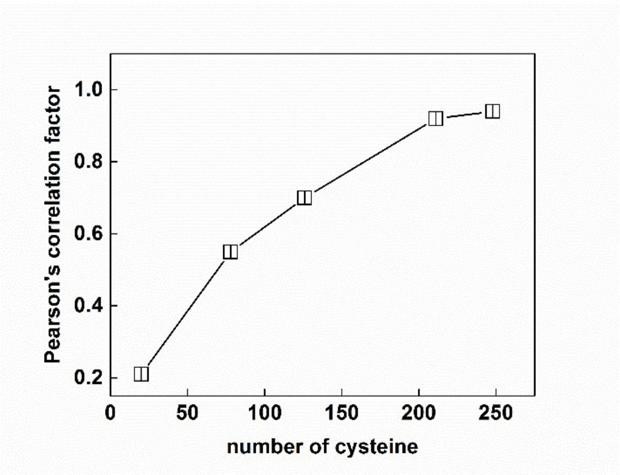

Figure S59. The relationship between the Golgi targeting ability and the number of cysteine on the surface of LC-CQDs. The Pearson's correlation factors were calculated after 4 h of incubation of HEp-2 cells with LC-CQDs (0.5 mg mL^{-1}).

Note: Golgi targeting ability would perform the best if the number of cysteine on carbon quantum dots is higher than 210. As shown in Figure S59, the Pearson's correlation factor between carbon quantum dots and Bodipy ceramide would increase as the increasing of cysteine number from 0 to 210. The Golgi targeting ability would not increase significantly when the number of cysteine is more than 210. In order to prevent oxidation during storage, excess cysteine molecules were linked on the surface of LC-CQDs in this work.

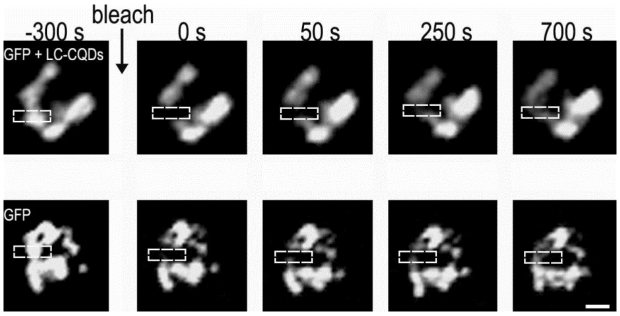

Figure S60. Fluorescence recovery of Golgi-GFP at 0-700 s after photobleaching of cells treated with or without LC-CQDs (1 mg mL^{-1}). Scale bar, 3 μm. The results suggest that Intra-Golgi trafficking is unaffected if the concentration of LC-CQDs is lower than 1 mg mL^{-1}.

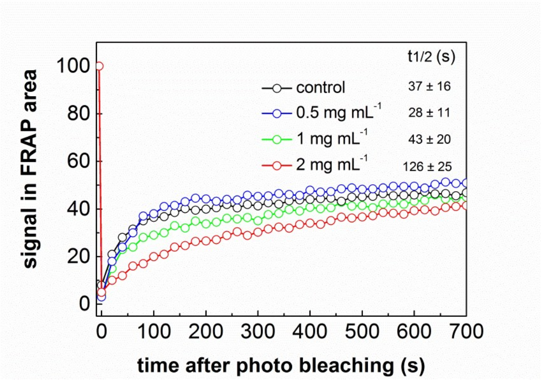

Figure S61. The fluorescence recovery plots of Golgi-GFP at 0-700 s after photobleaching of cells treated with different concentrations of LC-CQDs.

Note: The multivalent effect of LC-CQDs is investigated with the fluorescence recovery after photobleaching (FRAP) technique (Supplementary Figs 44 and 45). A patch of the Golgi co-stained with Golgi-GFP and LC-CQDs is selected for photobleaching. The $t_{1/2}$ value of the recovery of Golgi-GFP shows no significant difference, whether the cells are treated with typically applied concentrations of LC-CQDs (0.5 mg mL^{-1} and 1 mg mL^{-1}) or not. The $t_{1/2}$ value of the recovery is bigger than that of the control when cells are treated with a higher concentration of LC-CQDs (2 mg mL^{-1}), indicating decrease of the mobility of Golgi-GFP. Inhibition of Golgi-GFP mobility is not observed, when the Golgi is labeled with carbon quantum dots carrying no thiol groups. Consequently, mobility decrease should be attributed to the existence of multivalent effect of free thiol groups on the surface of LC-CQDs, which is responsible for the high Golgi specificity of LC-CQDs.

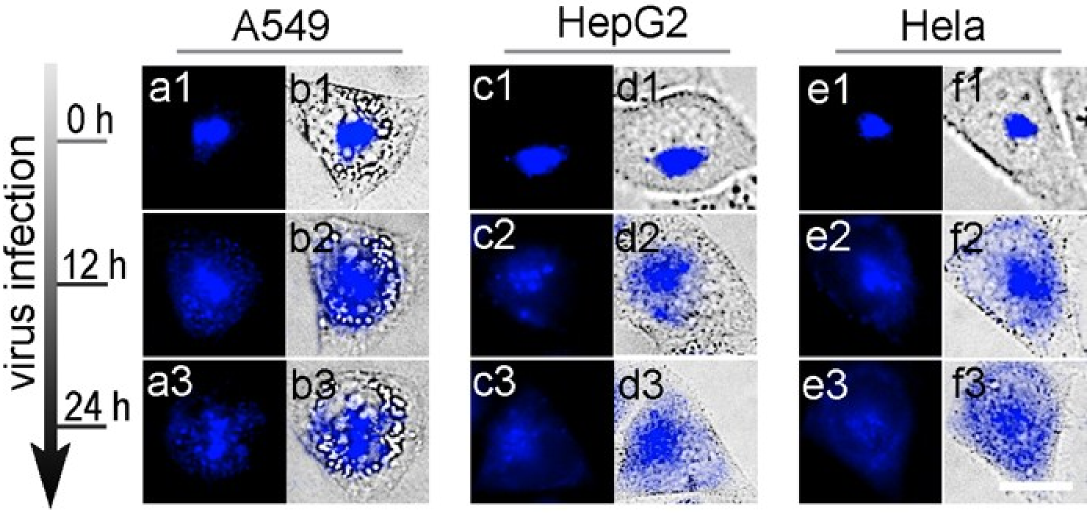

Figure S62. Morphological changes in the Golgi apparatus of A549, HepG2, and Hela cells during virus infection. (a1–a3) Fluorescence images of the Golgi stained with LC-CQDs in A549 cells. (b1–b2) Merged images of fluorescence and bright field images. (c1–c3) Fluorescence images of the Golgi stained with LC-CQDs in HepG2 cells. (d1–d3) Merged images of fluorescence and bright field images. (e1–e3) Fluorescence images of the Golgi stained with LC-CQDs in Hela cells. (f1–f3) Merged images of fluorescence and bright field images. Scale bar, 20 μm.

Note: To investigate whether the Golgi function deactivation induced by the virus infection is only for HEp2 cell line, we monitored the morphology changes of the Golgi stained by LC-CQDs in A549, HepG2, and Hela cells (Figure S62). Similar to the Hep2 cells, the morphology of the Golgi in A549, HepG2, and Hela cells would break into fragments and become scattered during the viral infection.

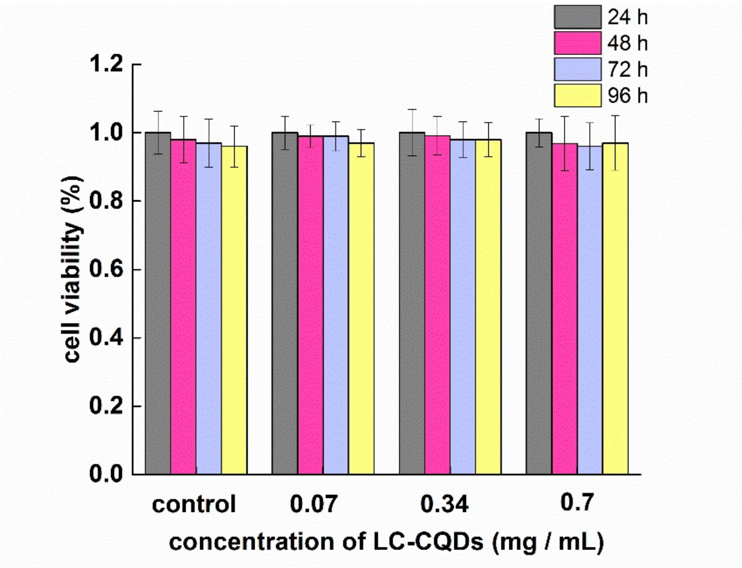

Figure S63. Cell viability of HEp2 cells with Cell Counting Kit-8. The viability of HEp2 cells was detected by incubating cells with LC-CQDs at different concentrations and for different time. The results indicate the good biocompatibility of LC-CQDs.

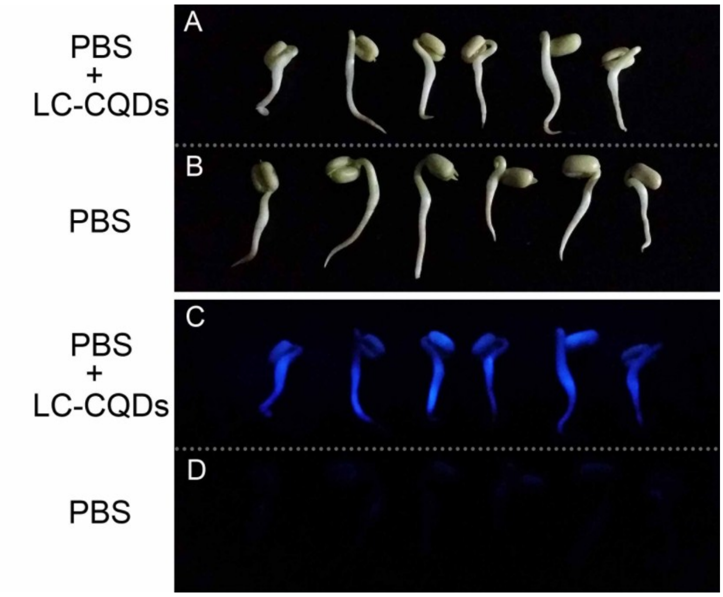

Figure S64. Fluorescence images of bean sprouts which grown with LC-CQDs aqueous solution (*A*, *C*) or PBS (*B*, *D*). Concentration: LC-CQDs, 1.5 mg / mL. **(***A* and *B***)** Under illumination of white light. **(***C* and *D***)** Under illumination of UV (365 nm) light. Notes: the optical images indicate that bean sprouts could grow normally in an aqueous solution of LC-CQDs. The fluorescence property of the sprouts indicates that LC-CQDs could permeate throughout cells, but were nontoxic and did not hinder plant growth. The results indicate the good biocompatibility of LC-CQDs.

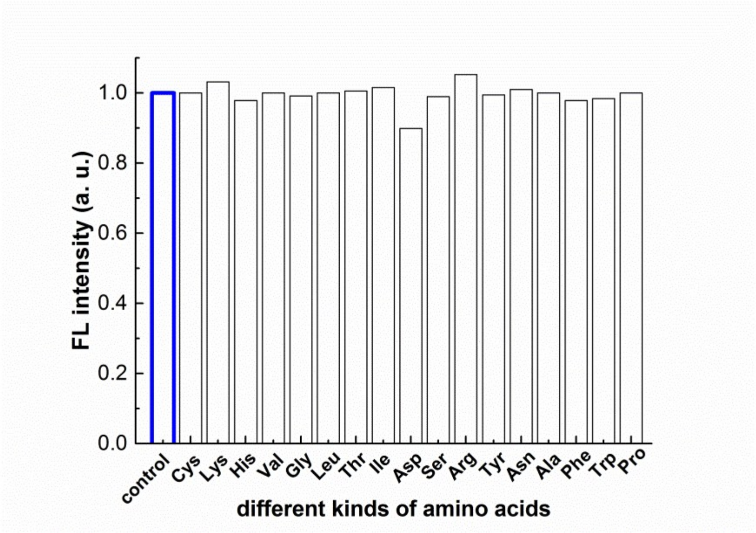

Figure S65. Effect of different amino acids on the fluorescence intensity of LC-CQDs. Concentration: LC-CQDs, 5 μg/mL; amino acids, 1 mM. Cys: cysteine; Lys: lysine; His: histidine; Val: valine; Gly: glycine; Leu: leucine; Thr: threonine; Ile: isoleucine; Asp: aspartic acid; Ser: serine; Arg: arginine; Tyr: tyrosine; Asn: asparagine; Ala:alanine; Phe: phenylalanine; Trp: tryptophan; Pro: proline.

Note: The influence of these amino acids to the fluorescence intensity should be examined before the experiment of cell imaging. The results indicate that the fluorescence intensity of LC-CQDs has not been influenced by these materials, demonstrating the possibility of LC-CQDs in the use of cell imaging.

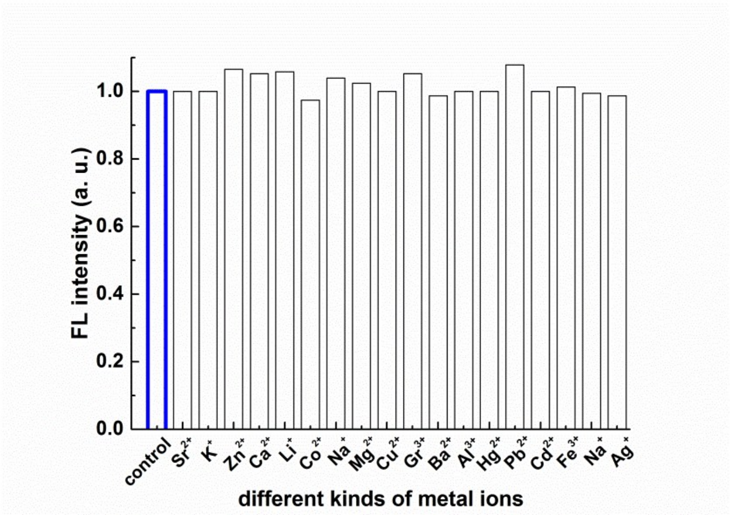

Figure S66. Effect of different metal ions on the fluorescence intensity of LC-CQDs. Concentration: LC-CQDs, 5 μg / mL; metal ions,1 μM. Notes: it is well know that all kinds of amino acids and metal ions are widely dispersed in cells.

Note: The influence of metal ions to the fluorescence intensity should be examined before the experiment of cell imaging. The results indicate that the fluorescence intensity of LC-CQDs has not been influenced by these materials, manifesting the possibility of LC-CQDs in the use of cell imaging.

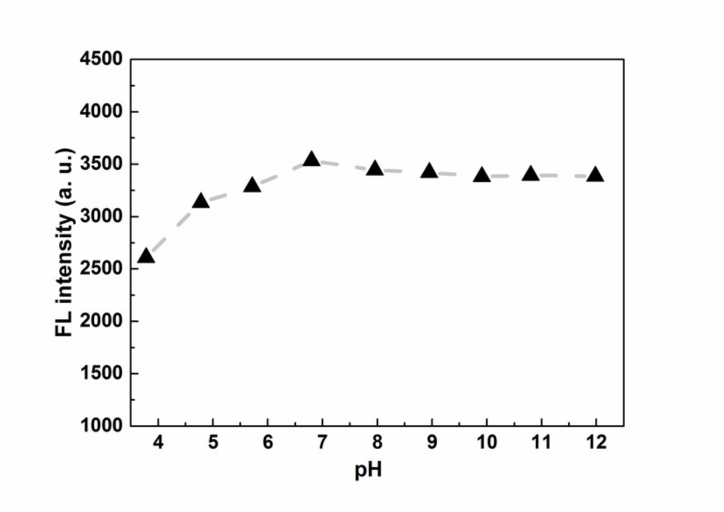

Figure S67. Effect of pH on the fluorescence intensity of LC-CQDs. The fluorescence intensity of LC-CQDs has not been influenced in the range of cellular pH. Concentration: LC-CQDs, 5 μg/mL.

Note: The influence of pH to the fluorescence intensity should be examined before the experiment of cell imaging. The results indicate that the fluorescence intensity of LC-CQDs is stable under physiological conditions, demonstrating the possibility of LC-CQDs in the use of cell imaging.

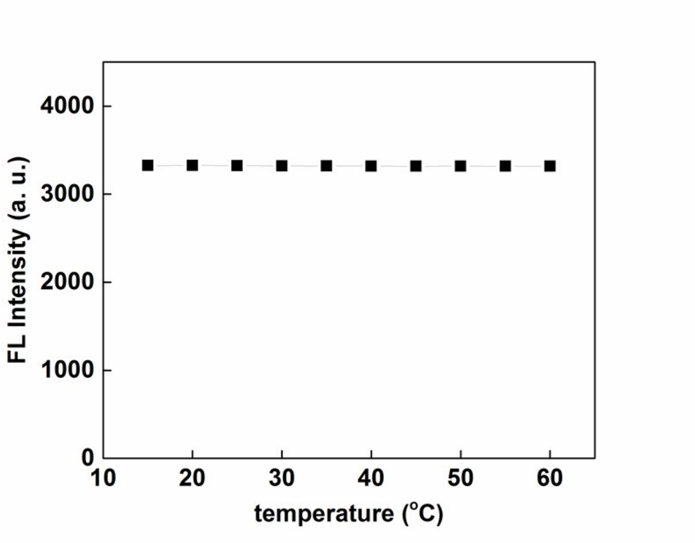

Figure S68. Effect of temperature on the fluorescence intensity of LC-CQDs. The fluorescence intensity of LC-CQDs has not been influenced from 15 ^{o}C to 60 ^{o}C. Concentration: 5 μg/mL.

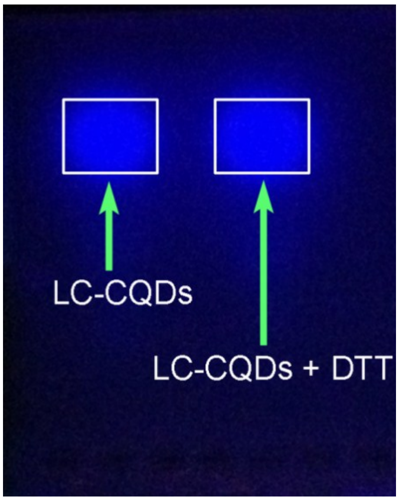

Figure S69. The results of agarose gel electrophoresis of LC-CQDs and LC-CQDs + DTT. The blue bands are the fluorescence of LC-CQDs. 2% w / v agarose gel, 3 volt / cm.

Note: Agarose gel electrophoresis and Ellman's test were used for investigating whether disulfide was formed between the adjacent cysteines on the carbon quantum dots. Dithiothreitol (DTT) is a reducing agent, which can proceed the reduction of a typical disulfide bond by two sequential thiol-disulfide exchange reactions. LC-CQDs reacted with or without DTT were used in the agarose gel electrophoresis and Ellman's test. LC-CQDs and LC-CQDs + DTT may be separated by charge through agarose gel electrophoresis if disulfide was formed on the LC-CQDs. However, there is no obvious difference between the fluorescence band of LC-CQDs and LC-CQDs + DTT (Figure S69), indicating few disulfide bonds may form between the adjacent cysteines on the carbon quantum dots. Furthermore, Ellman's reagent (DTNB) used in Scheme 1 was adopted to detect the amount of thiol groups of LC-CQDs reacted with or without DTT. It is need to note that we had purified the LC-CQDs after the reaction with DTT. The amount of thiol groups of LC-CQDs reacted with DTT is not higher than the amount of thiol groups of LC-CQDs reacted without DTT. The results of agarose gel electrophoresis and Ellman's test suggest that disulfide may not form between the adjacent cysteines on the as-prepared carbon quantum dots.

The cysteine residues were kept the reduced state by adding excess cysteine (~1.4 g) in the synthesis process. The carbon quantum dots were wrapped in a lot of cysteine molecules. Cysteine molecules near carbon quantum dots could bind on the surface of carbon quantum dots through condensation of amino groups with carboxyl groups. The excess cysteine can make carbon quantum dots isolated from oxygen and scavenge reactive oxygen species generated in the synthesize process. The as-prepared carbon quantum dots were dissolved in water without oxygen and stored at -20 ^{o}C.

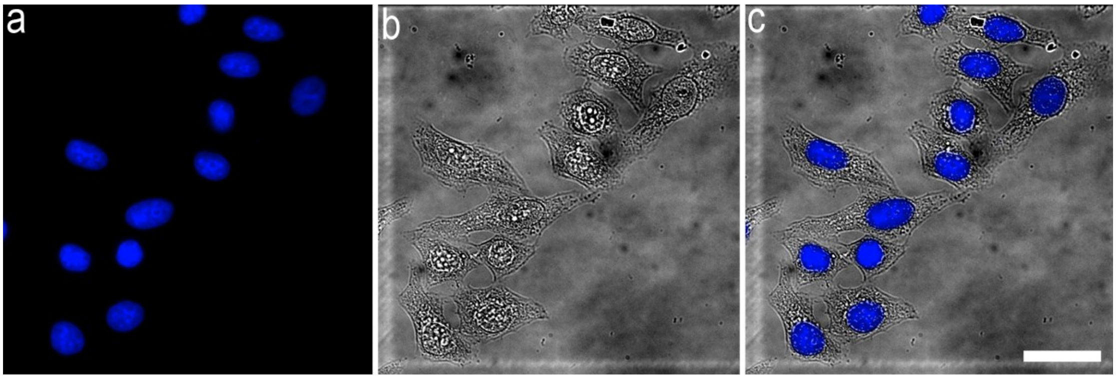

Figure S70. The cells are not contaminated by mycoplasma. (**a**) Fluorescence image of Hoechst; DNA was stained with Hoechst. No small bright extranuclear dots or rods were observed. (**b**) Bright field image of the HEp2 cells. (**c**)

Merged images of fluorescence and bright field images. Scale bar, 20 µm. The images are representative of replicate experiments (n = 5).

Note: HEp-2 cells were tested for mycoplasma contamination by fluorochrome DNA staining test. HEp-2 cells were cultured in a 35 mm glass-bottom dish for over 24 h. Then the cells were fixed with 4% paraformaldehyde for 20 min and washed with PBS for three times. After that, the cells were incubated with 1 µg mL^{-1} Hoechst 33258 stain for 10 min and rinsed. The dish was transferred to an Olympus IX-81 microscope for fluorescence imaging. Hoechst 33258 was excited at 360-370 nm and detected with BA420-460 nm. If the cells were contaminated with mycoplasma, the small bright extranuclear dots or rods would be observed. The tests were done five times before any experiment. No small bright extranuclear dots or rods were observed, suggesting that the cells were not contaminated by mycoplasma (Supplementary Fig. 51).

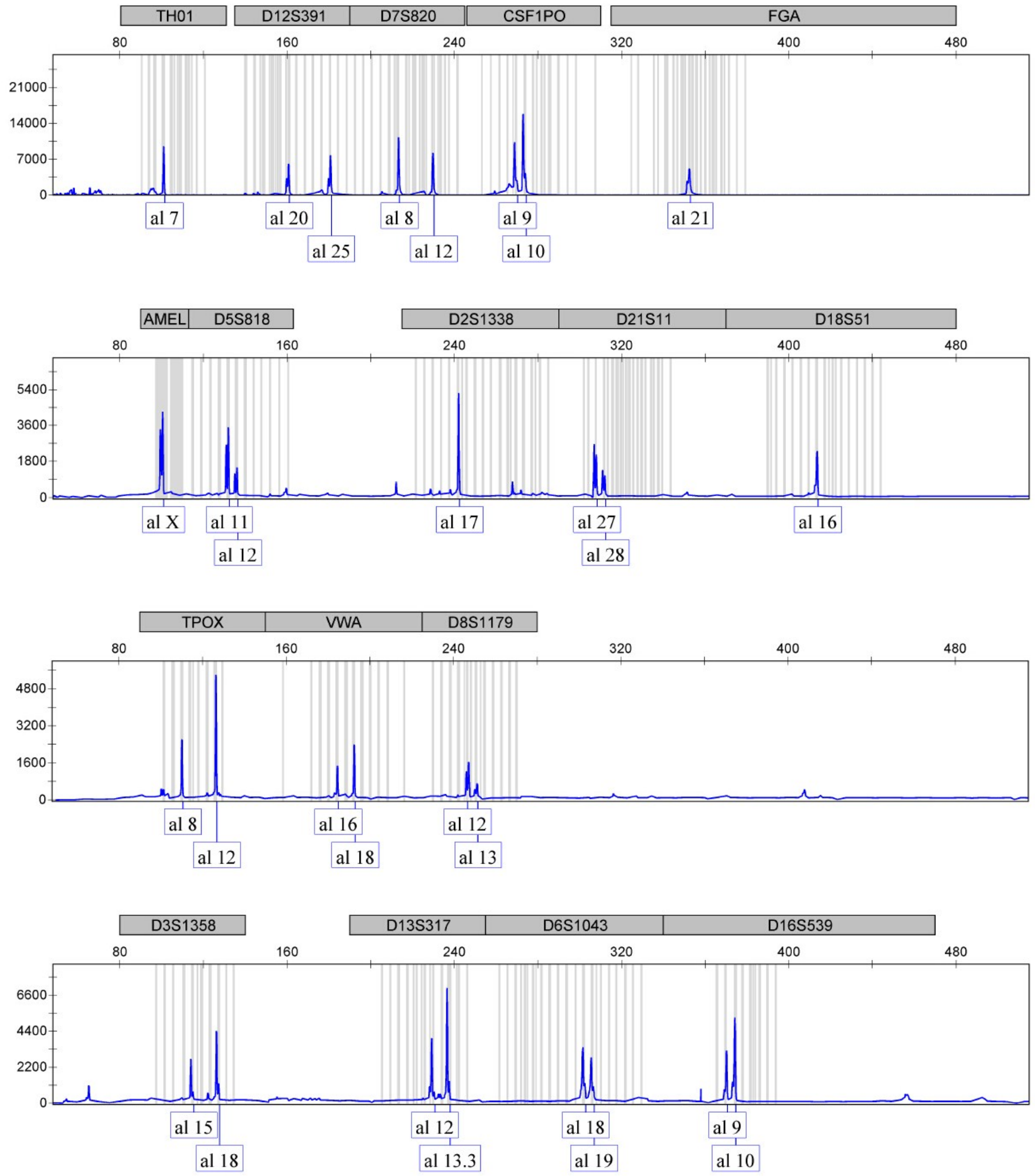

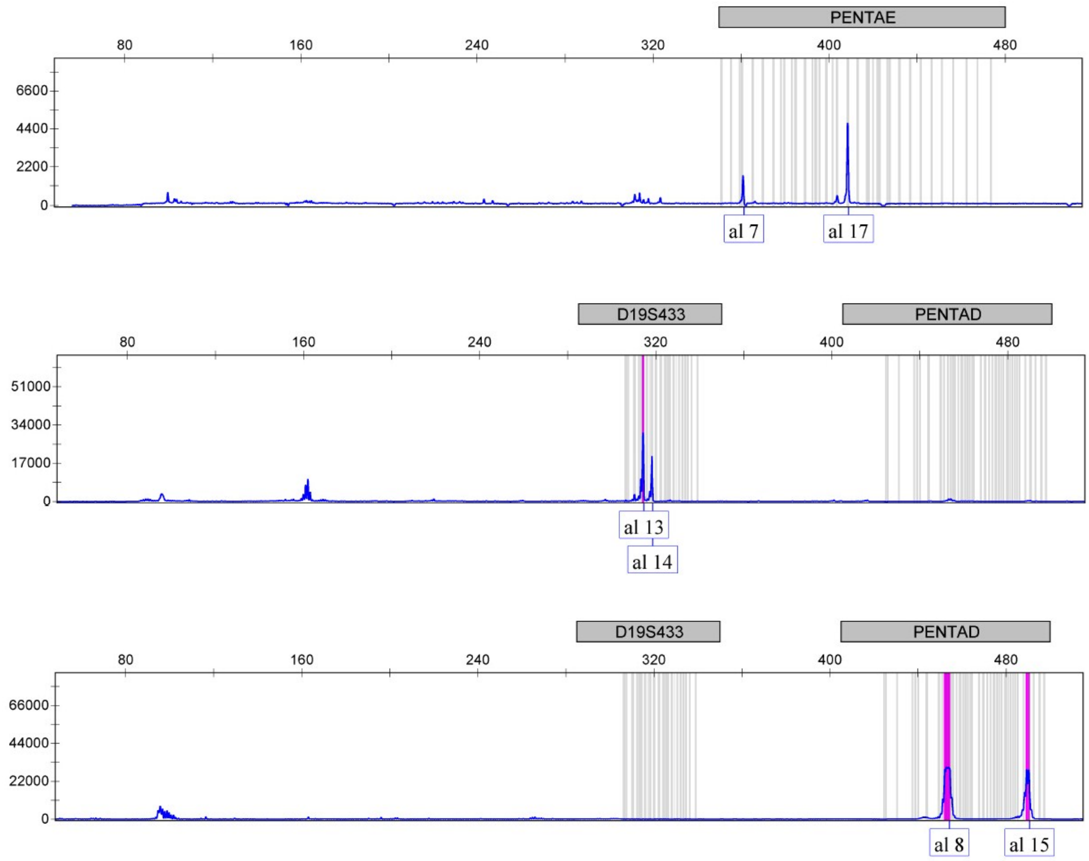

Figure S71. Results of Short Tandem Repeat (STR) profiling of Hep2 cells used in this experiment. Images are representative of replicate experiments (n = 3).

Antibody information

Anti- EEA1 antibody was purchased from abcam. Rabbit polyclonal antibody to EEA1 – Early Endosome Marker. Catalog number: ab2900. Tested species reactivity: Human, Mouse, Rat, Chicken, Hamster, Cow, Dog, Xenopus laevis, Zebrafish, Rhesus, Monkey, Aplysia. Immunogen: Synthetic peptide derived from within residues 1350 to the C-terminus of Human EEA1. Anti-Rab11 antibody was validated by Western blot analysis (Supplementary Fig. 53a). This product has been referenced (PubMed: 25295779 and 25486514).

Anti-Rab9 antibody was purchased from abcam. Rabbit monoclonal antibody to Rab9 – Late endosome marker. Catalog number: ab179815. Clone number: EPR13272. Tested species reactivity: Human, Rat, Mouse. Immunogen: Synthetic peptide within Human Rab9 aa 1-100 (Cysteine residue). The exact sequence is proprietary. Anti-Rab9 antibody was validated by Western blot analysis (Supplementary Fig. 53b).

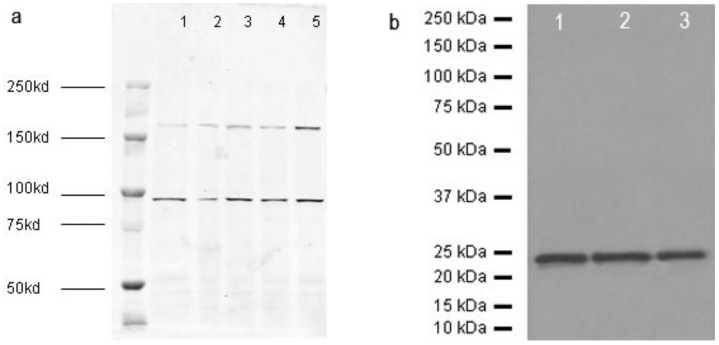

Figure S72. Western blot - EEA1 antibody and Western blot – Rab9 antibody. (**a**) Anti-EEA1 antibody - Early Endosome Marker (ab2900) at 1/1000 dilution. Lane 1 : HeLa nuclear. Lane 2 : HeLa whole cell lysate. Lane 3 : A431 cell lysate. Lane 4 : Jurkat cell lysate. Lane 5 : HEK293 cell lysate. Lysates/proteins at 20 µg per lane. Secondary: Alexa Fluor anti rabbit at 1/50000 dilution. Performed under reducing conditions. (**b**) Western blot - Anti-Rab9 antibody [EPR13272] - Late Endosome Marker (ab179815). All lanes : Anti-Rab9 antibody [EPR13272] - Late Endosome Marker (ab179815) at 1/2000 dilution (purified). Lane 1 : K562 cell lysate. Lane 2 : HeLa cell lysate. Lane 3 : HepG2 cell lysate. Lysates/proteins at 20 µg per lane. Secondary: HRP goat anti-rabbit IgG (H+L) at 1/1000 dilution. Predicted band size : 23 kDa. Observed band size : 23 kDa. Blocking buffer: 5% NFDM/TBST. Dilution buffer: 5% NFDM/TBST.

Anti-Rab11 antibody was purchased from abcam. Rabbit polyclonal antibody to Rab11. Catalog number: ab3612. Tested species reactivity: Human, Dog. Immunogen: Synthetic peptide corresponding to Human Rab11 aa 1-17; Sequence: MGTRDDEYDYLFKVVLIC. Anti-Rab11 antibody was validated by Western blot analysis (Supplementary Fig. 54a). This product has been referenced (PubMed: 22613725 and 18495814).
Anti-GM130 antibody was purchased from abcam. Rabbit monoclonal antibody to GM130 – cis-Golgi marker. Catalog number: ab52649. Clone number: EP892Y. Tested species reactivity: Human, Cow, Dog, Monkey, Africa Green Monkey. Immunogen: Synthetic peptide (the amino acid sequence is considered to be commercially sensitive) corresponding to Human GM130 aa 1-100. Anti-Rab9 antibody was validated by Western blot analysis (Supplementary Fig. 54b). This product has been referenced (PubMed: 26934330 and 24649404).
Anti-TGN46 antibody was purchased from abcam. Rabbit polyclonal antibody to TGN46. Catalog number: ab50595. Tested species reactivity: Human. Immunogen: synthetic peptide, KASDYQRLDQKY. Conjugated to KLH by a N-terminal Cysteine residue linker, corresponding to amino acids 426-437 of Human TGN46. Anti-Rab11 antibody was validated by Western blot analysis. Use a concentration of 0.25 - 0.5 µg/ml. Detects a band of approximately 80-100 kDa. This product has been referenced (PubMed: 25208761 and 21385839).
Goat Anti-Rabbit IgG H&L (Cy3®) was purchased from abcam. Goat polyclonal antibody secondary antibody to Rabbit IgG - H&L (Cy3®), pre-adsorbed. Catalog number: ab6939. Tested species reactivity: Rabbit. Immunogen: full length native Rabbit IgG (purified). Conjugate: Cy3®. Ex: 552 nm, Em: 565 nm. This product was validated by Immunocytochemistry/ Immunofluorescence - Histone H3 (acetyl K9) antibody and has been referenced (PubMed: 26052937 and 26656251).

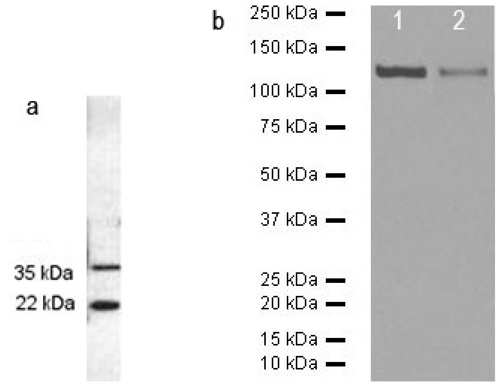

Figure S73. Western blot – Rab11 antibody and Western blot – GM130 antibody. (**a**) Western blot - Rab11 antibody (ab3612). Predicted band size : 24 kDa. ab3612 used on PC3 cell extract. (**b**) Western blot - Anti-GM130 antibody [EP892Y] - cis-Golgi Marker (ab52649). Anti-GM130 antibody [EP892Y] - cis-Golgi Marker (ab52649) at 1/1000 dilution (purified). Lane 1 : HeLa cell lysate. Lane 2 : MCF7 cell lysate. Lysates/proteins at 20 µg per lane. Secondary: Peroxidase-conjugated goat anti-rabbit IgG, (H+L) at 1/1000 dilution.
